# Taxonomic revision of Neotropical *Murdannia* Royle (Commelinaceae)

**DOI:** 10.3897/phytokeys.74.9835

**Published:** 2016-11-08

**Authors:** Marco Octávio de Oliveira Pellegrini, Robert B. Faden, Rafael Felipe de Almeida

**Affiliations:** 1 Universidade de São Paulo, Departamento de Botânica, Rua do Matão 277, CEP 05508-900, São Paulo, SP, Brazil; 2 Jardim Botânico do Rio de Janeiro, Rua Pacheco Leão 915, CEP 22460-030, Rio de Janeiro, RJ, Brazil; 3 Smithsonian Institution, NMNH, Department of Botany, MRC 166, P.O. Box 37012, Washington D.C. 20013-7012, USA; 4 Universidade Estadual de Feira de Santana, Programa de Pós-Graduação em Botânica, Avenida Transnordestina s/n, Novo Horizonte, CEP 44036-900, Feira de Santana, BA, Brazil

**Keywords:** Aquatic plants, Brazil, Commelinales, Commelineae, dewflower, Neotropical flora, spiderwort

## Abstract

This study provides a taxonomic revision for the Neotropical species of the genus *Murdannia*. Six species are recognized as native, including a new species and a new combination, while two Asian species are recognized as invasive. We present an identification key, a table summarizing the morphologic differences among the species, a new synonym, six lectotypifications, a distribution map, and descriptions, comments and photographic plates for each species. We also provide comments on the morphology of the Neotropical species of *Murdannia*, comparing them with the Paleotropical species, and a discussion of inflorescence architecture in the genus as a whole.

## Introduction

*Murdannia* Royle is one of the largest genera in Commelinaceae, comprising ca. 60 species (Faden 1998; eMonocot 2010; The Plant List 2013; Govaerts and Faden 2016). It was described by Royle (1840), based on *Aneilema scapiflorum* Roxb. [= *Murdannia edulis* (Stokes) Faden], and was named after Murdan Ali, the keeper of the Saharanpur Botanic Garden herbarium at India. Unware of Royle’s name, Brückner (1926) described *Phaeneilema* G.Brückn., and transferred several species from *Aneilema* to his new genus (Brückner 1926, 1927). A few years later, in his treatment for Commelinaceae in Engler’s *Natürlichen Pflanzenfamilien*, Brückner (1930) realized that *Phaeneilema* and *Murdannia* were congeneric and made the required combinations. Pichon (1946) pointed out the existence of two names prior to *Murdannia* (i.e. *Dilasia* Raf. and *Streptylis* Raf.), both published by Rafinesque (1838) in his Flora Telluriana. Since both names were published in the same work and none had priority over the other, *Dilasia* was adopted by Pichon (1946) as the accepted name. Nevertheless, Brenan (1952), noted that most of the necessary combinations in *Murdannia* had already been made by Brückner (1930). Thus, the author proposed to conserve *Murdannia* against *Dilasia* and *Streptylis*, to avoid the unnecessary creation of about 30 new combinations in the monospecific *Dilasia* (Brückner 1930; Merrill 1937).

*Murdannia* nom. cons. is currently placed in subfamily Commelinoideae, tribe Commelineae, together with *Aneilema* R.Br., *Buforrestia* C.B.Clarke, *Commelina* L., *Floscopa* Lour., *Pollia* Thunb., and *Stanfieldiella* Brenan, among others (Faden and Hunt 1991; Evans et al. 2003; Burns et al. 2011; Faden 1998). As aforementioned, *Murdannia* species have been historically treated under *Aneilema* by several authors (e.g. Brown 1810; Clarke 1881; Bentham and Hooker 1883; Woodson 1942), and sometimes also under *Commelina*. Nevertheless, *Murdannia* can be easily differentiated from *Aneilema* and *Commelina* by its flowers commonly enantiostylous, petals sessile and equal to subequal, three antesepalous stamens (one sometimes staminodial), three antepetalous staminodes, 3-lobed antherodes, capsules always equally 3-locular, and 3-valved (Brenan 1952, 1966; Faden 1998). The genus has a Pantropical and Warm Temperate distribution, being especially diverse in Asia, where most (more than 50%) of the accepted species and morphological diversity are known to occur (Nandikar 2013; Ancy 2014; Ancy and Nampy 2014; Nandikar and Gurav 2015). Most recent studies on *Murdannia* have focused on the Paleotropical species, especially the Asian (e.g. Faden 2001; Nandikar 2013; Ancy 2014; Ancy and Nampy 2014; Nandikar and Gurav 2015) and the African (Faden 2012) members of the genus. Nevertheless, very little is known about the Neotropical species of the genus (Pellegrini et al. 2013). A total of four Neotropical species of *Murdannia* were accepted in the most recent account on the group (Barreto 1997; eMonocot 2010), with the occurrence of *Murdannia schomburgkiana* (Kunth) G.Brückn. in Brazil being considered doubtful. Barreto (1997), in her unpublished Ph.D. thesis and based on the limited material she had available, also considered the *Murdannia gardneri* (Seub.) G.Brückn. species complex to be composed of a sole and widely polymorphic and distributed species. In the most recent checklist for the Brazilian Flora (BFG 2015), Barreto’s taxonomic viewpoints were followed in detail, with the sole addition of *Murdannia nudiflora* (L.) Brenan as an invasive species.

Recent field and herbaria studies have shed some light in this neglected group. As a first attempt to clarify the taxonomy and systematics of Neotropical Commelinaceae, the present study provides a revision of the Neotropical species of *Murdannia*, with the description of a new species (endemic to Central-Western Brazil), and a new combination. We also provide a detailed taxonomic treatment on the group and comments on the morphology and systematics of *Murdannia* as a whole.

## Methods

The descriptions and phenology of the species were based on herbaria, spirit, fresh material and literature. Descriptions of *Murdannia engelsii* M.Pell. & Faden, sp. nov., *Murdannia nudiflora* and *Murdannia paraguayensis* (C.B.Clarke ex Chodat) G.Brückn. were complemented, using spirit samples kindly provided by the collectors, and living samples. Specimens from the following herbaria were also analyzed: ALCB, B, BHCB, BHZB, BM, BRIT, C, CEPEC, CESJ, CNMT, CVRD, ESA, F, FCAB, FLOR, FURB, G, GH, GUA, HAMAB, HAS, HB, HBR, HERBAM, HRB, HSTM, HUEFS, HURB, IAC, ICN, INPA, K, MBM, MBML, MG, MO, MY, NY, P, PORT, R, RB, RFA, RFFP, SP, SPF, TANG, TCD, UEC, UPCB and US (herbaria acronyms according to Thiers, continuously updated). The distribution of the species is based on herbarium materials, field data and literature. The classification of the vegetation patterns follows IBGE (2012). The indumenta and shapes terminology follows Radford et al. (1974); the inflorescence terminology and morphology follows Weberling (1965, 1989) and Panigo et al. (2011); the fruit terminology follows Spjut (1994) and Ancy and Nampy (2014); and seeds terminology follows Faden (1991) and Ancy and Nampy (2014). The conservation statuses were proposed following the recommendations of *IUCN Red List Categories and Criteria, Version 3.1* (IUCN 2001). GeoCAT (Bachman et al., 2011) was used for calculating the Extent of Occurrence (EOO) and the Area of Occurrence (AOO). The generic description of *Murdannia* presented in this work applies only to the Neotropical region, and is not meant to reflect the entire morphological diversity of this widespread and diverse genus.

## Results

In the present work, we accept six species native to the Neotropical region, with a new combination and a new species, and recognize two invasive Asian species. We present below descriptions for all native species, detailed diagnosis for the two invasive species, and a table summarizing the morphologic differences between all species found in the Neotropical region (Table 1). We also provide comments on the morphology of the Neotropical species of *Murdannia*, comparing them with the Paleotropical species, and a discussion of inflorescence architecture in the genus as a whole.

**Table 1.** Morphologic characters differentiating the species of *Murdannia* known for the Neotropical region.

Characters	*Murdannia burchellii*	*Murdannia engelsii*	*Murdannia gardneri*	*Murdannia nudiflora*	*Murdannia paraguayensis*	*Murdannia schomburgkiana*	*Murdannia semifoliata*	Murdannia aff. triquetra
**Phyllotaxy**	Spirally-alternate	Distichously-alternate	Spirally-alternate	Distichously-alternate	Spirally-alternate, sometimes becoming distichously-alternate at apex	Spirally-alternate	Spirally-alternate	Spirally-alternate
**Inflorescence**	Terminal or axillary in the uppermost nodes; pedunculate	Terminal or axillary in the uppermost nodes; pedunculate	Terminal or axillary in the uppermost nodes; pedunculate	Terminal or axillary in the uppermost nodes; pedunculate	Terminal or axillary in the uppermost nodes; pedunculate	Mainly axillary; sessile	Mainly axillary; sessile	Mainly axillary; sessile
**Cincinnus bracts**	Cup-shaped, apex caudate	Flat, apex acute	Cup-shaped, apex acuminate	Cup-shaped, apex acute	Flat, apex acute	Tubular, apex truncate	Tubular, apex truncate	Not observed
**Cincinni**	(1–)2–16, alternate to sub-opposite, 2–9-flowered	1, solitary, 2–7-flowered	16–38, verticillate, 2–11-flowered	1, solitary, 2–12-flowered	9–24, verticillate, 1-flowered	1–2–(3), fascicle-like, 1-flowered	1–2–(3), fascicle-like, 1-flowered	1–2–(3), fascicle-like, 1-flowered
**Floral buds**	Narrowly ovoid to ovoid	Ovoid	Narrowly ovoid to ovoid	Ellipsoid to oblongoid	Narrowly ovoid	Ellipsoid	Ellipsoid	Ellipsoid
**Flower symmetry**	Enantiostylous	Enantiostylous	Enantiostylous	Zygomorphic	Enantiostylous	Actinomorphic	Actinomorphic	Actinomorphic?
**Petals pubescence**	Glabrous	With minute glandular hairs at base on the adaxial surface	Glabrous	Glabrous	With minute glandular hairs at base on the adaxial surface	Densely bearded with moniliform hairs on the adaxial surface	Densely bearded with moniliform hairs on the adaxial surface	Glabrous
**Filaments pubescence**	Glabrous	With glandular hairs	Glabrous	Bearded with moniliform hairs	With glandular hairs	Bearded with moniliform hairs	Bearded with moniliform hairs	Not observed
**Anthers**	Narrowly elliptic to narrowly oblong, connective lilac, anthers sacs white	Elliptic, connective white to lilac, anthers sacs white to light-lilac	Elliptic, connective lilac to white, anthers sacs white to lilac	Elliptic to oblong, connective bluish-lilac to white, anthers sacs purple to dark-purple	Elliptic to oblong, connective purple to bluish-purple, anthers sacs lilac to purple	Elliptic to oblong, connective brown, anthers sacs brownish-lilac	Linear-oblong to oblong, connective purple, anthers sacs lilac to purple	Not observed
**Antherodes**	Sagittate, golden yellow	Subsagittate to subcordate, golden-yellow	Cordate, golden yellow	Hastate, white to cream	Sagittate, golden yellow	Hastate, golden yellow	Hastate, golden yellow	Not observed
**Gynoecium pubescence**	Glabrous	With glandular hairs	Glabrous	Glabrous	With glandular hairs	Glabrous	Glabrous	Glabrous
**Fruiting pedicel**	Erect	Deflexed	Erect	Erect	Deflexed	Erect	Erect	Apparently erect
**Capsules**	Subglobose to globose	Broadly ovoid to broadly ellipsoid	Subglobose to globose	Ovoid to subglobose	Oblongoid to broadly oblongoid	Oblongoid to broadly oblongoid	Oblongoid to broadly oblongoid	Oblongoid to ellipsoid
**Seeds**	1 per locule, reniform to broadly ellipsoid, ventri-lateral appendage present	1 per locule, reniform to broadly ellipsoid, ventri-lateral appendage present	1 per locule, reniform to broadly ellipsoid, ventr -lateral appendage present	2 per locule, broadly ellipsoid to oblongoid, ventri-lateral appendage absent	2 per locule, reniform to broadly-ellipsoid, ventri-lateral appendage present	6 per locule, cuboid to polygonal, ventri-lateral appendage absent	6 per locule, cuboid to polygonal, ventri-lateral appendage absent	3 per locule, transversely ellipsoid, ventri-lateral appendage absent

### Murdannia

Taxon classificationPlantaeCommelinalesCommelinaceae

Royle, Ill. Bot. Himal. Mts. 1: 403, pl. 95, f. 3. 1839.

Aphylax Salisb., Trans. Hort. Soc. London 1: 271. 1812, nom. nud. Type species. Aphylax spiralis (L.) Salisb. [≡ Murdannia spirata (L.) G.Brückn.].
Baoulia A.Chev., Bull. Soc. Bot. France 58 (8): 217. 1912. Type species. Baoulia tenuissima A.Chev. [≡ Murdannia tenuissima (A.Chev.) Brenan].
Dichaespermum Wight, Icon. Pl. Ind. Orient. 6: 31. 1853. Type species (designated here). Dichaespermum lanceolatum Wight [≡ Murdannia lanceolata (Wight) Kammathy].
Dilasia Raf., Fl. Tellur. 4: 122. 1838, nom. rej. Type species. Dilasia vaginata (L.) Raf. [≡ Murdannia vaginata (L.) G.Brückn.].
Ditelesia Raf., Fl. Tellur. 3: 69. 1837 nom. rej. Type species. Ditelesia nudiflora (L.) Raf. [≡ Murdannia nudiflora (L.) Brenan].
Phaeneilema G.Brückn., Bot. Jahrb. Syst. Beibl. 137: 63. 1926, nom. illeg. Type species. Phaeneilema sinicum (Ker Gawl.) G.Brückn. [=Murdannia simplex (Vahl) Brenan]
Prionostachys Hassk., Flora 49: 212. 1866. Type species (designated here). Prionostachys ensifolia Hassk. ex C.B. Clarke [= Murdannia gigantea (Vahl) G.Brückn.].
Streptylis Raf., Fl. Tellur. 4: 122. 1838, nom. rej. Type species. Streptylis bracteolata Raf. [= Murdannia spirata (L.) G.Brückn.].
Talipulia Raf., Fl. Tellur. 2: 17. 1837, nom. rej. Type species. Talipulia malabarica (L.) Raf. [= Murdannia nudiflora (L.) Brenan].

#### Type species.

*Murdannia scapiflora* (Roxb.) Royle [= *Murdannia edulis* (Stokes) Faden].

#### Description.

*Herbs*, perennial or annual, rhizomatous or not, with a definite or indefinite base, terrestrial to paludal to rooted emergent aquatics. *Roots* thin and fibrous or tuberous and fusiform. *Rhizomes* short to elongate. *Stems* trailing and ascending at the apex or erect, unbranched to densely branched, rooting in the rhizome and at the basal nodes, rarely at the distal ones when they touch the substrate. *Leaves* sessile; distichously or spirally-alternate, congested at the apex of the stem or evenly distributed along the stem; lamina flat to slightly falcate to falcate and/or conduplicate, base symmetrical, midvein inconspicuous to conspicuous, adaxially impressed or not, abaxially prominent or not, secondary veins conspicuous to inconspicuous. *Synflorescence* composed of a solitary main florescence or with 1–several coflorescences. *Main florescences (inflorescences)* terminal or axillary in the in the uppermost nodes, not perforating the leaf-sheaths; main florescence a thyrse, composed of 1–many cincinni; basal bract reduced to leaf-like; peduncle bracts (sterile bracts) absent; cincinni bracts persistent; cincinni, sessile to pedunculate, contracted to elongate, bracteoles flat or tubular, persistent or caducous. *Flowers* bisexual or male (the male ones with a reduced gynoecium), actinomorphic, zygomorphic or enantiostylous, chasmogamous, flat (not tubular); pedicels erect at anthesis and pre-anthesis, erect or deflexed at post-anthesis; sepals 3, equal, free, cucullate, membranous to chartaceous, dorsally not keeled, margins hyaline, accrescent and persistent in fruit; petals 3, sessile, equal to subequal, free, deliquescent, glabrous or with minute glandular hairs at base or medially bearded with moniliform hairs on the adaxial surface; stamens (2–)3, equal, antesepalous, filaments bent ca. 30° either to the left or to the right, free, glabrous or with minute glandular hairs or medially bearded with moniliform hairs, anthers dorsifixed, rimose, connective narrow, anther sacs parallel, elongate; staminodes 3–(4), antepetalous (if 4 staminodes are present, than 1 antesepalous to the lower sepal), filaments free, glabrous, minutely glandular-puberulous basally or medially bearded with moniliform hairs, antherodes dorsifixed, 3-lobed, indehiscent, connective expanded, golden yellow or mauve to purple; ovary sessile, bent ca. 30° on the opposite direction as the stamens, smooth, glabrous or glandular-puberulous, 3-locular, locules equal, locule 1–2–(6)-ovulate, style erect or gently curved at the apex, stigma truncate to capitate, papillate. *Capsules* loculicidal, 3-valved, apiculate due to persistent style base, smooth, glabrous or glandular-puberulous. *Seeds* exarillate, farinose, uniseriate, 1–2–(6) per locule, reniform to broadly ellipsoid or cuboid to polygonal, slightly to strongly cleft towards the embryotega, ventrally flattened or not, testa costate to slightly rugose or shallowly scrobiculate to scrobiculate to foveolate, with ridges radiating from the embryotega, appendaged or not, hilum elliptic or linear, embryotega lateral to semilateral or semidorsal.

#### Ecology and habitat.

As with most aquatic plants, Neotropical *Murdannia* are seldom collected throughout their distribution range. Despite that, they seem to be locally common or uncommon, depending on the species. They all seem to be intimately related to permanent and seasonal water bodies of drier domains and vegetation, such as flooded grasslands in the Cerrado, Chaco and Pantanal domains, or the white sand formations in the Amazon basin.

#### Morphological relationships among the Neotropical species and within the genus.

*Murdannia* is one of the six (i.e. *Aneilema*, *Buforrestia*, *Commelina*, *Floscopa* and *Pollia*) out of 41 genera of Commelinaceae distributed in the Neotropics and Paleotropics. (Faden 1998). Although few in number, the Neotropical species of *Murdannia* exhibit all the extremes in inflorescence morphology found in *Murdannia* as a whole. The terminal thyrse consisting of well-spaced whorls of cincinni, present in *Murdannia gardneri* and *Murdannia paraguayensis*, is elsewhere present only in the rare Central African *Murdannia allardii* (De Wild.) Brenan, and in the Asian species *Murdannia divergens* (C.B.Clarke) G.Brückn, and *Murdannia juncoides* (Wight) R.S.Rao & Kammathy (Ancy 2014; Faden & Pellegrini pers. obs.). Glandular-pubescent sepals and pedicels, present in *Murdannia burchellii* (C.B.Clarke) M.Pell., comb. et stat. nov., *Murdannia englesii*, *Murdannia gardneri*, and *Murdannia paraguayensis*, are otherwise known only from the Asian species *Murdannia medica* (Lour.) D.Y.Hong (usually present) and *Murdannia spectabilis* (Kurz) Faden. Moniliform hairs on the upper surface of the petals, present in *Murdannia schomburgkiana* (Kunth) G.Brückn. and *Murdannia semifoliata* (C.B.Clarke) G.Brückn., are recorded only in the Asian and African *Murdannia simplex* (Vahl) Brenan. One-seeded capsule locules, which characterize *Murdannia burchellii*, *Murdannia engelsii* and *Murdannia gardneri*, are known only in the Asian/Malaysian *Murdannia vaginata* (L.) G.Brückn., and in the Indian *Murdannia assamica* Nampy & A.Ancy (Ancy and Nampy 2014). Finally, characters present in one or more Neotropical species that are not recorded elsewhere in the genus, include: (1) inflorescences with whorls of 1-flowered cincinni (present in *Murdannia paraguayensis*); (2) the presence of glandular hairs on the inflorescence axis, cincinnus peduncles and axes (present in *Murdannia burchellii*, *Murdannia englesii*, *Murdannia gardneri*, and *Murdannia paraguayensis*); (3) petals with minute glandular hairs at base on the adaxial surface (present in *Murdannia engelsii* and *Murdannia paraguayensis*); (4) the presence of glandular hairs on the filaments, ovaries and capsules (present in *Murdannia engelsii* and *Murdannia paraguayensis*); (5) long moniliform hairs on the petals and not confined to the petal bases (present in *Murdannia schomburgkiana* and *Murdannia semifoliata*); and (6) appendages on the seeds (present in *Murdannia burchellii*, *Murdannia engelsii*, *Murdannia gardneri* and *Murdannia paraguayensis*).

#### Inflorescence architecture in *Murdannia*.

Brenan (1966) has shown a great diversity of inflorescence architecture in *Murdannia*, with variations in the position of the main florescence, total number of cincinni, number of nodes with cincinni, number of cincinni per node, and degree of development of each cincinnus. According to Panigo et al. (2011), the basic inflorescence pattern for Commelinaceae is a many-branched, pedunculate and terminal thyrse, with verticillate cincinni, each cincinnus multi-flowered. Based on Brenan (1966) and Panigo et al. (2011), we could also infer that the plesiomorphic inflorescence architecture for *Murdannia* would correspond to the basic inflorescence pattern for Commelinaceae. Brenan (1966) indicates that most of the variation in inflorescence architecture could be derived from this basic type, as exemplified by the Asian *Murdannia divergens*, by only three changes. On the other hand, Panigo et al. (2011) states that additional changes would be necessary to express all the known variation in the inflorescence morphology for *Murdannia*, as: (1) the production of coflorescences, in addition to the main florescence; (2) variation in the length of the peduncle and internodes of the main florescence; (3) variation in the number of cincinni per node; (4) variation in the arrangement of cincinni on each node of the main florescence; (5) variation in the length of the cincinnus peduncle; and (6) variation in the total flower number per cincinnus. These changes can occur separately or in different combinations. In the most extreme cases, the inflorescences are mainly axillary, each being fascicle-like, and composed of a few 1-flowered cincinni.

If we were to consider this stepwise change a possible evolutionary sequence within *Murdannia*, then the South American species with the most plesiomorphic inflorescence type would be *Murdannia gardneri*. By its reduced number of cincinni per node and change in their arrangement, the inflorescence of *Murdannia burchellii* could be morphologically derived from *Murdannia gardneri*. *Murdannia paraguayensis*, shares the numerous verticillate cincinni of *Murdannia gardneri*, but each cincinnus is reduced to a single flower. *Murdannia engelsii* has terminal or terminal and axillary inflorescences, that are reduced to single cincinni, but the cincinnus is 2–several-flowered. The most reduced inflorescences, and perhaps the ones that accumulated the greatest number of stepwise changes, can be observed in *Murdannia schomburgkiana* and *Murdannia semifoliata*, in which most inflorescences are fascicle-like, axillary in the distal leaves, and with all cincinni 1-flowered. Species with similarly reduced inflorescences are numerous in Asia [e.g. *Murdannia blumei* (Hassk.) Brenan, *Murdannia crocea* (Griff.) Faden, *Murdannia keisak* (Hassk.) Hand.-Mazz., *Murdannia lanuginosa* (Wall. ex C.B.Clarke) G.Brückn., *Murdannia pauciflora* (Wight) G.Brückn., *Murdannia triquetra* (Wall. ex C.B.Clarke) G.Brückn., and *Murdannia versicolor* (Dalzell) G.Brückn.], and represented in Africa by *Murdannia axillaris* Brenan (Faden 2012; Ancy 2014). Nonetheless, some of them show characters not present in any of the Neotropical species, such as annual habit, biseriate seeds and yellow to orange flowers. Thus, in the absence of a well sampled molecular phylogeny it would be impossible to state whether the Neotropical species represent one or several distinct lineages in *Murdannia*.

#### Key to the native and invasive species of *Murdannia* in the Neotropics

**Table:** 

1	Inflorescences composed of 2–several verticillate or alternate to subopposite cincinni, rarely composed of a solitary cincinnus, bracteoles persistent; flowers enantiostylous, sepals with glandular hairs or with a mixture of glandular and eglandular hairs, androecium glabrous or with minute glandular hairs; seeds with a ventri-lateral appendage	**2**
–	Inflorescences composed of a solitary cincinnus or fascicle-like, bracteoles caducous; flowers non-enantiostylous, sepals glabrous, androecium medially bearded with moniliform hairs; seeds without a ventri-lateral appendage	**5**
2	Bracteoles cup-shaped; pedicels erect at post-anthesis and in fruit; petals glabrous, filaments, ovaries and capsules glabrous; hilum in a deep depression	**3**
–	Bracteoles flat; pedicels deflexed at post-anthesis and in fruit; petals with minute glandular hairs at base on the adaxial surface, filaments, ovaries and capsules with glandular hairs; hilum in a shallow depression	**4**
3	Cincinni alternate, rarely subopposite, sinuate; plants generally delicate; stems prostrate, thin, densely branched at the base; leaves chartaceous, linear to linear-oblong; main axis of inflorescence with sparse eglandular and glandular hairs; cincinnus bracts with caudate apex; seeds densely farinose, the testa costate to slightly rugose	***Murdannia burchellii* (C.B.Clarke) M.Pell.** (Fig. 1)
–	Cincinni verticillate, straight; plants generally robust; stems ascending to erect, succulent, little branched at base to unbranched; leaves succulent, linear-lanceolate to lanceolate; main axis of inflorescence with dense glandular and sparse eglandular hairs; cincinnus bracts with acuminate apex; seeds farinose, the testa scrobiculate to foveolate	***Murdannia gardneri* (C.B.Clarke) G.Brückn.** (Figs 3–4)
4	Inflorescence reduced to a solitary cincinnus (but sometimes several clustered in a synflorescence near towards the shoot apex), peduncles with a mixture of eglandular (scabrid) and glandular to densely glandular hairs, cincinni 2–7-flowered; plants without a definite base; leaves distichously-alternate; flowers buds ovoid; capsules broadly ovoid to broadly ellipsoid, locules 1-seeded	***Murdannia engelsii* M.Pell. & Faden** (Fig. 2)
–	Inflorescence a terminal thyrse composed of several whorls of 1-flowered cincinni, peduncles with glandular to densely glandular hairs, cincinni 1-flowered; plants with a definite base; leaves spirally-alternate; flower buds ellipsoid to narrowly ellipsoid; capsules oblongoid to broadly oblongoid, locules 2-seeded	***Murdannia paraguayensis* (C.B.Clarke ex Chodat) G.Brückn.** (Fig. 6)
5	Leaves distichously-alternate; inflorescences long-pedunculate, exerted from the leaf-sheaths, cincinni 2–12-flowered, pendent; flowers zygomorphic, stamens 2, staminodes 4 (1 staminode antesepalous, sometimes lacking the antherode), antherodes white to cream; capsules ovoid to subglobose	***Murdannia nudiflora* (L.) Brenan** (Fig. 5)
–	Leaves spirally-alternate; inflorescences sessile, enclosed by the leaf-sheaths; cincinni 1-flowered, erect; flowers actinomorphic, stamens 3, staminodes 3, antherodes yellow (flowers uncertain in Murdannia aff. triquetra); capsules oblongoid to ellipsoid	**6**
6	Annuals without a definite base; roots thin; stems trailing, apex ascending, densely branched; petals glabrous; capsules with 3-seeded locules; seeds transversely ellipsoid	**Murdannia aff. triquetra (Wall. ex C.B.Clarke) G.Brückn.** (Fig. 9)
–	Perennials with a definite base; roots tuberous; stems erect (only the short rhizome prostrate), unbranched; petals medially bearded with moniliform hairs on the adaxial surface; capsules with 6-seeded locules; seeds cuboid to polygonal	**7**
7	Leaf-blades margins glabrous throughout, inflorescences-bearing leaves with expanded blades (2.2–13.6 cm long); anthers brown	***Murdannia schomburgkiana* (Kunth) G.Brückn.** (Fig. 7)
–	Leaf-blades margins ciliate at least at base, inflorescences-bearing leaves reduced to bladeless sheaths or with much reduced blades (0.2–1.8 cm long); anthers purple	***Murdannia semifoliata* (C.B.Clarke) G.Brückn.** (Fig. 8)

**Figure 1.**
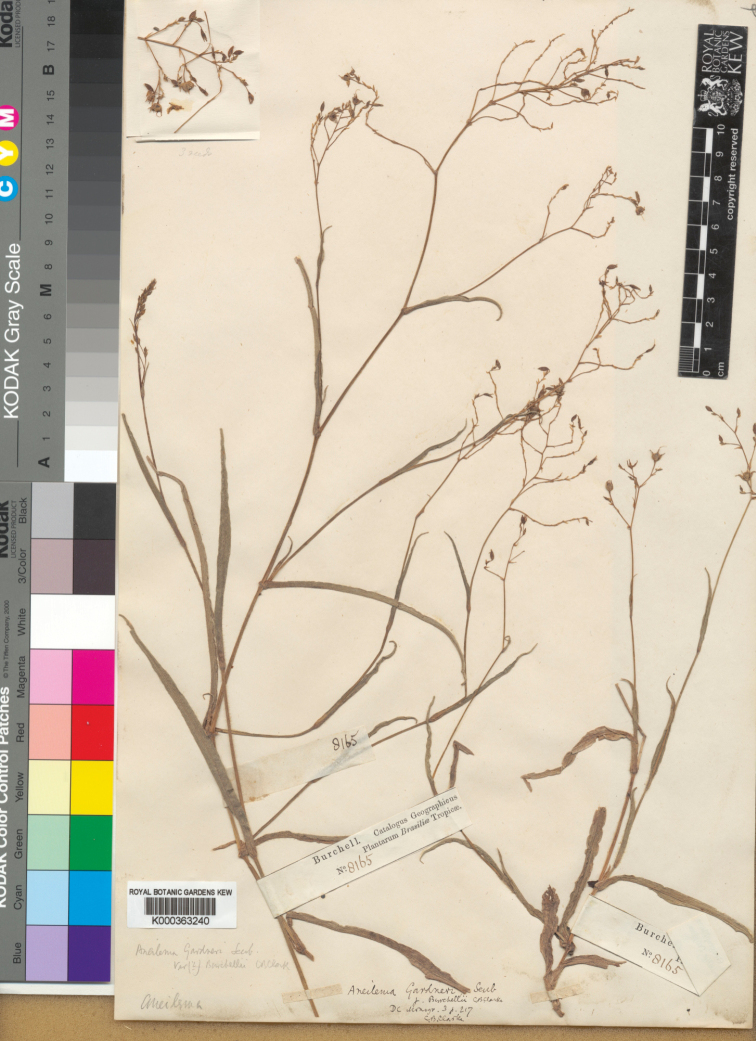
*Murdannia burchellii* (C.B.Clarke) M.Pell. Lectotype of *Aneilema gardneri* var. *burchellii* (K barcode K000363240). Photograph courtesy of Royal Botanic Gardens, Kew, London.

**Figure 2.**
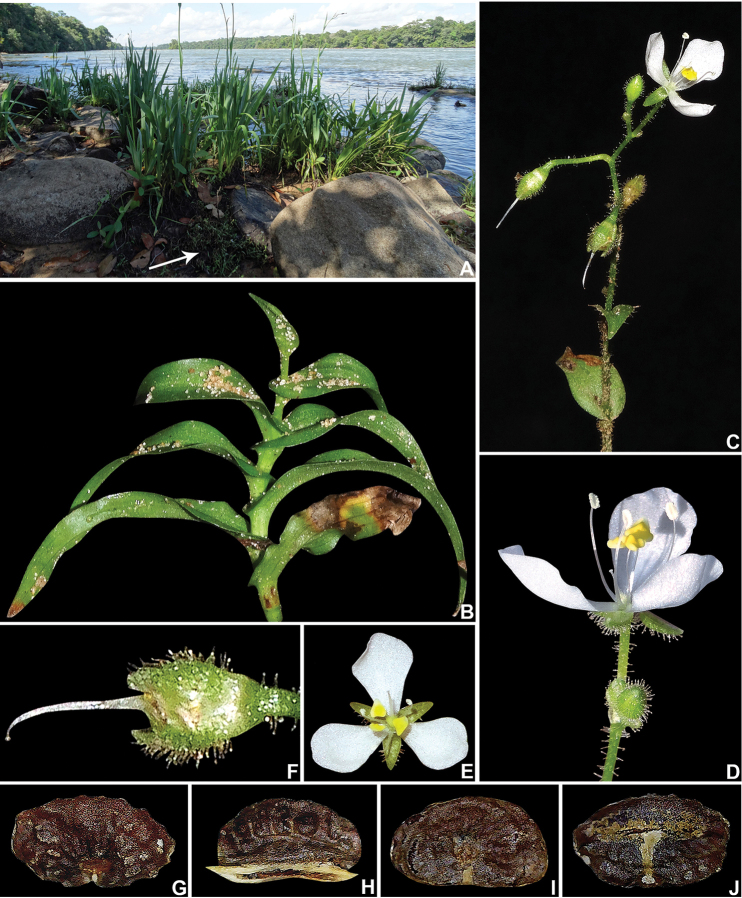
*Murdannia engelsii* M.Pell. & Faden. **A** Sandy banks of rio Teles Pires, white arrow showing a subpopulation of *Murdannia engelsii* **B** detail of the stem, showing the conduplicate and falcate leaves, with amplexicaul bases **C** detail of the inflorescence, showing the deflexed pedicels at post-anthesis **D** side view of a male flower, showing the short and bent style. **E** front view of a bisexual flower, showing the long curved style **F** detail of a young fruit, showing the pedicel and sepals with glandular hairs, gently curved style and capitate stigma **G–J** seeds: **G** dorsal view of a seed, showing the scrobiculate and cleft testa, and the semilateral embryotega **H** ventral view of the same seed, showing the ventral furrows and tan appendage surrounding the hilum **I** dorsal view of another seed, showing the shallowly scrobiculate and slightly cleft testa, and the semidorsal embryotega **J** ventral view of the same seed, with the appendage removed, showing the linear hilum in a shallow depression. K, dorsal view of a seed, showing the smooth testa. Photographs **A–F** by M.E. Engels, **G–J** by R.F. Almeida.

**Figure 3.**
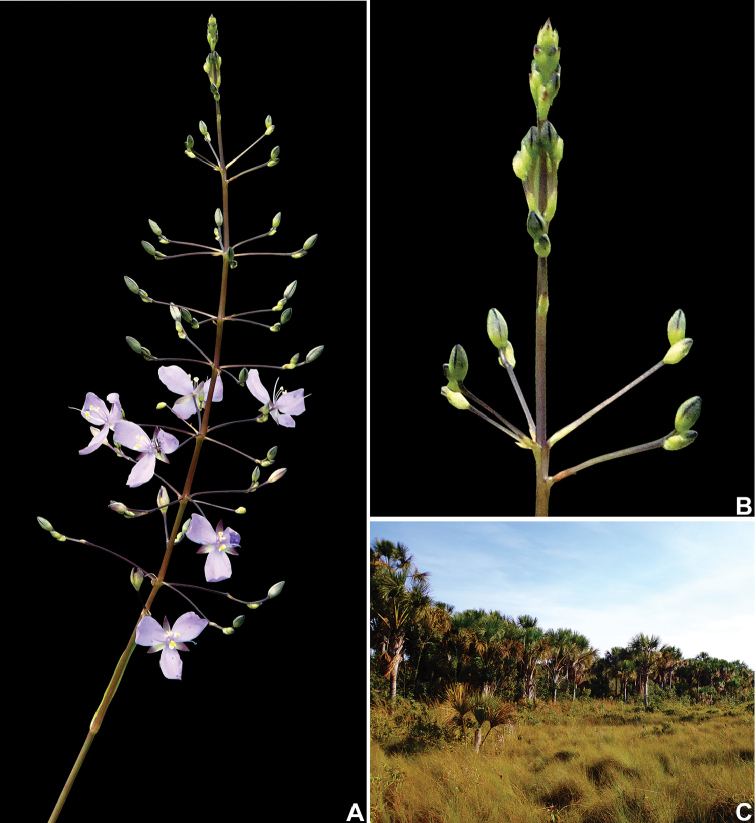
*Murdannia gardneri* (Seub.) G.Brückn. **A** Inflorescence, showing the verticillate cincinni and open lilac flowers **B** detail of the inflorescence, showing the ascending and straight cincinni **C** flooded grassland in the state of Minas Gerais. Photographs **A–B** by W. Milliken, **C** by I.L.M. Resende.

**Figure 4.**
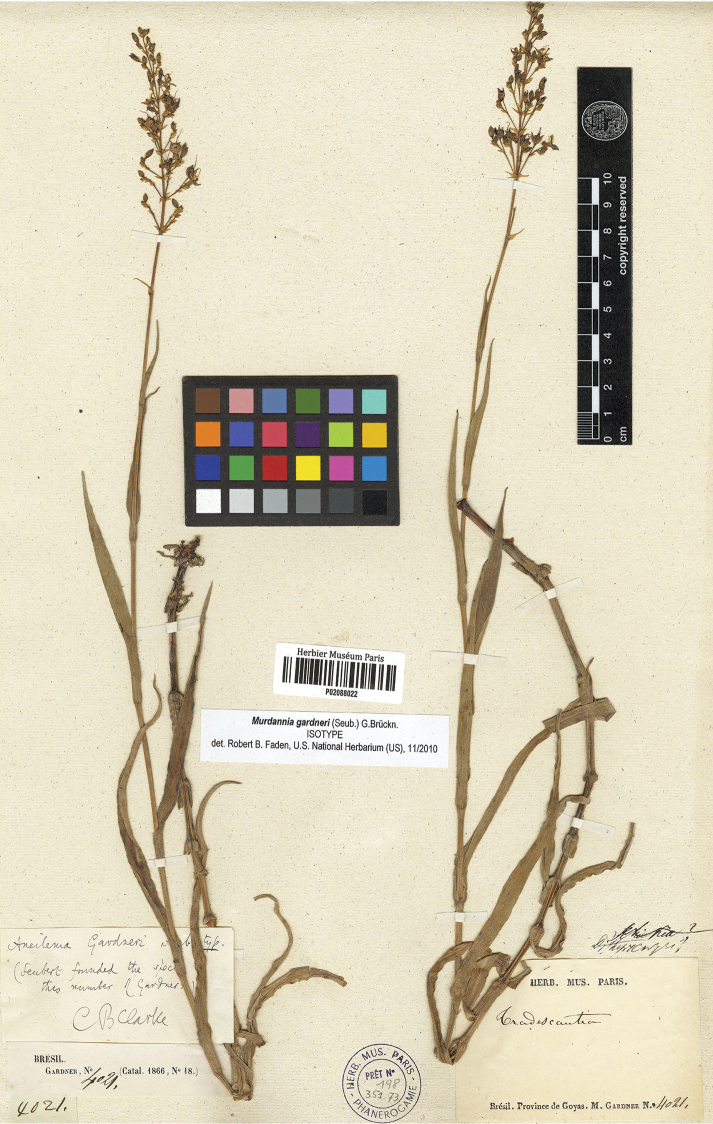
*Murdannia gardneri* (Seub.) G.Brückn. Isolectotype of *Aneilema gardneri* (P barcode P02088022). Photograph courtesy of the Muséum National d’Histoire Naturelle, Paris.

**Figure 5.**
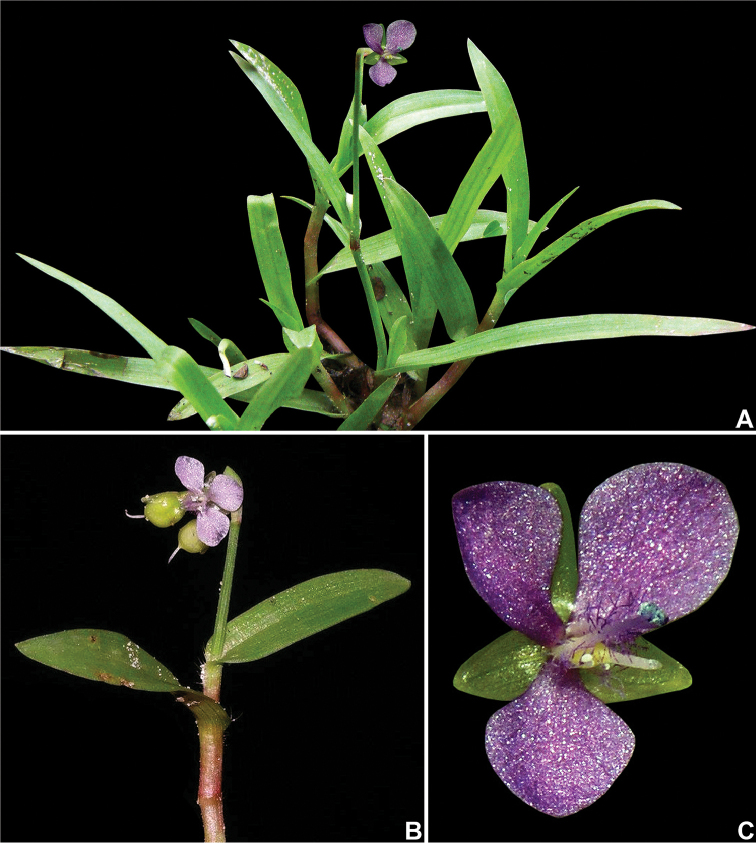
*Murdannia nudiflora* (L.) Brenan. **A** Habit **B** detail of a stem, showing the apical and long-pedunculate inflorescence **C** front view of a bisexual flower. Photograph **A, C** by W. Vargas and **B** by M.E. Engels.

**Figure 6.**
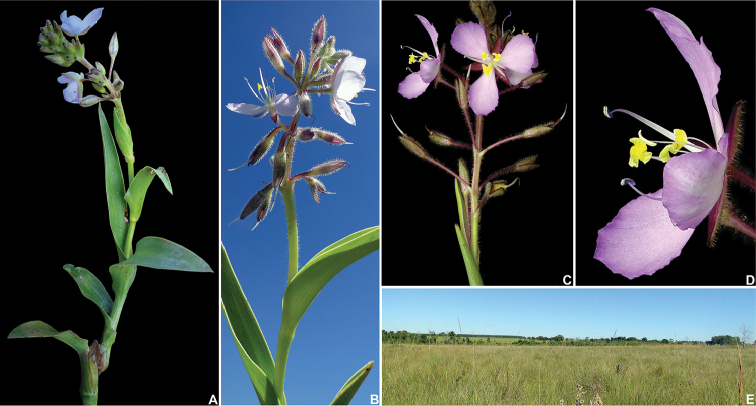
*Murdannia paraguayensis* (C.B.Clarke) G.Brückn. **A** Detail of a flowering shoot, showing the succulent stem, succulent, canaliculate and falcate leaves, and an inflorescence with lilac flowers **B** Detail of the apex of a flowering shoot, showing a terminal inflorescence with white flowers, and pedicels deflexed post-anthesis **C** Inflorescence showing the 1-flowered verticillate cincinni and open mauve flowers **D** Side view of a male flower, showing the sepals with glandular hairs **E** flooded grassland in Sidrolândia, Mato Grosso do Sul. Photograph **A** by I.L.M. Resende, **B, E** by S.N. Moreira and **C–D** by V.C. Souza.

**Figure 7.**
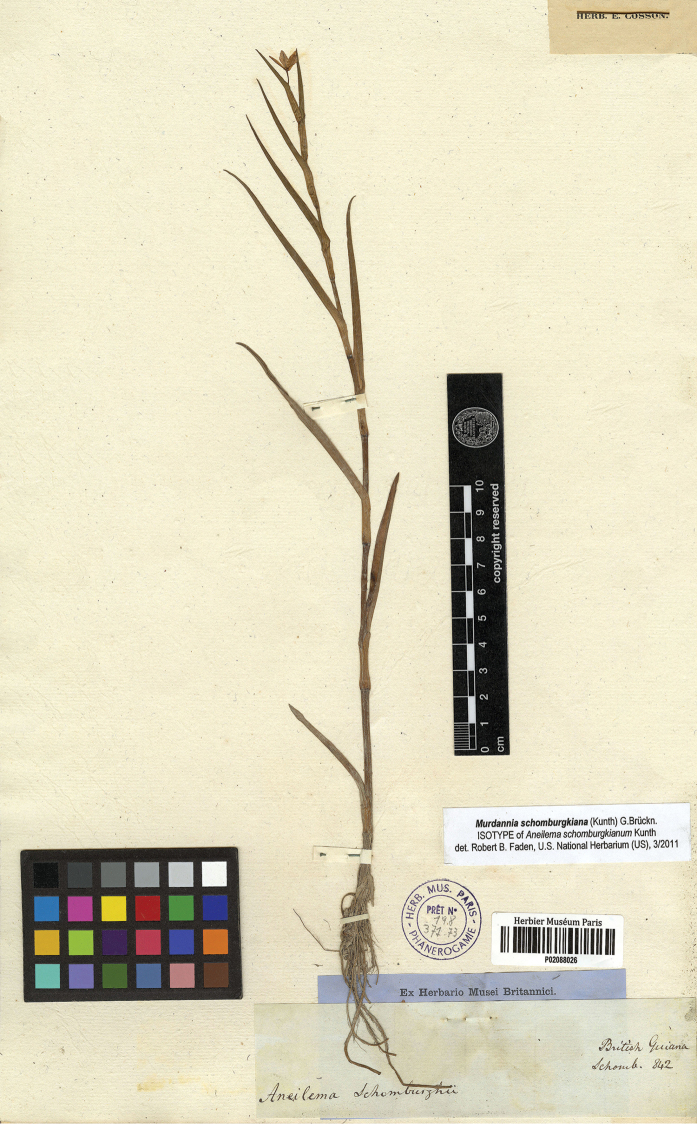
*Murdannia schomburgkiana* (Kunth) G.Brückn. Isolectotype of *Aneilema schomburgkianum* (P barcode P02088026). Photograph courtesy of the Muséum National d’Histoire Naturelle, Paris.

**Figure 8.**
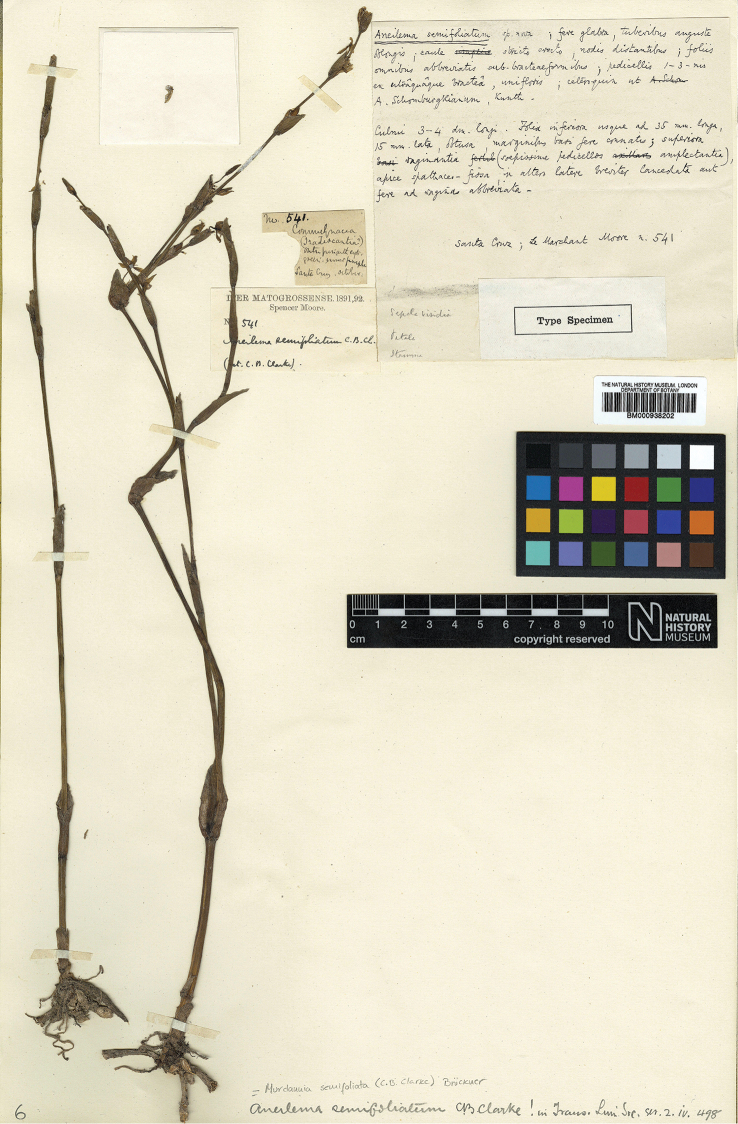
*Murdannia semifoliata* (C.B.Clarke) G.Brückn. Lectotype of *Aneilema semifoliatum* (BM barcode BM000938202). Photograph courtesy of The Natural History Museum of London.

**Figure 9.**
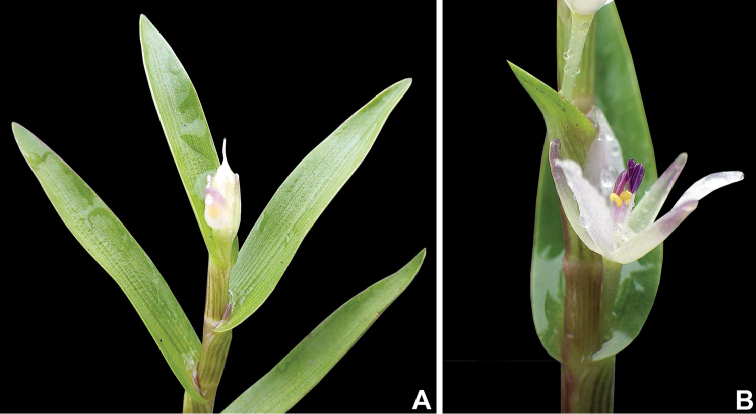
*Murdannia* aff. *triquetrum* (Wall. ex C.B.Clarke) G.Brückn., from Esteros de Arauca, Colombia. **A** Detail of a stem, showing an apical fruit **B** detail of an internode, showing a side view of a male flower. Photographs by M. Fernández.

### 1. Murdannia burchellii

Taxon classificationPlantaeCommelinalesCommelinaceae

(C.B.Clarke) M.Pell. comb. et stat. nov.

urn:lsid:ipni.org:names:77158526-1

Figs 1 , 10

Aneilema gardneri var. burchellii C.B.Clarke, Monogr. Phan. 3: 217. 1881. Lectotype (designated here): BRAZIL. s.loc., fl., fr., s.dat., W.J. Burchell 8165 (K barcode K000363240!; isolectotypes: GH barcode GH00415446!, P barcode P02088020!).
Aneilema gardneri var. glabrior C.B.Clarke, Monogr. Phan. 3: 217. 1881. Lectotype (designated here): BRAZIL. Goyaz, fl., fr., 1841, G. Gardner 4020 (P barcode P02088023!; isolectotypes: BM not found, G barcode G00098263!, NY barcode NY00247402!). **Syn. nov.**

#### Description.

*Herbs* ca. 14.0–55.0 cm tall., perennial, rhizomatous with a definite base, terrestrial to paludal to rooted emergent in flooded fields. *Roots* thin, fibrous, brown to dark-brown, densely to sparsely pilose with medium to dark brown hairs, emerging from the rhizome and from the basal most nodes. *Rhizomes* short, light to medium brown, buried in the sand or ground. *Stems* trailing with ascending apex, thin, densely branched or branched only at the base; internodes 1.8–8.4 cm long, green to vinaceous to reddish brown, sparsely pilose to hispid with hyaline hairs, becoming glabrous with age, with a line of eglandular hyaline hairs opposite the leaf above. *Leaves* spirally-alternate, evenly distributed along the stems, the distal ones gradually smaller than the proximal ones; sheaths 0.3–1.3 cm long, vinaceous to reddish brown, sparsely pilose to hispid with hyaline hairs, becoming glabrous with age, hairs hyaline, margins setose, with a line of eglandular hairs opposite to the leaf above; lamina 2.7–13 × 0.3–0.6 cm, linear to linear oblong, membranous, conduplicate, slightly falcate, light green to greyish green on both sides, drying light brown to olive-green on both sides, sparsely pilose to hispid, becoming glabrous with age, rarely glabrous, base truncate, margins green, ciliate to setose throughout or only at base, apex acuminate to mucronate; midvein conspicuous, impressed adaxially, prominently acute abaxially, secondary veins 2–(3) pairs, adaxially inconspicuous to slightly conspicuous, dark green, abaxially somewhat conspicuous, dark green. *Inflorescences* 1–2–(4) thyrsi, terminal or axillary in the uppermost nodes, thyrse with (1–)2–16, alternate to subopposite cincinni; peduncles 2.3–7.6 cm, with a sparse mixture of eglandular (scabrid) and glandular, hyaline hairs; basal bract reduced or leaf-like, 1.4–5.1 × 0.1–0.3 cm, lanceolate to linear, sparsely pilose to hispid, rarely glabrous, base truncate, margins ciliate to setose, apex acuminate, veins inconspicuous, concolorous or green; cincinni bracts ca. 0.2–1.1 × 0.1–0.4 cm, triangular to broadly triangular, cup-shaped, light green to lilac, glabrous to pilose at base, base amplexicaul, non-perfoliate, margins glabrous to sparsely ciliate, apex caudate; cincinni 2–9-flowered, erect, sinuate, cincinnus peduncle 0.4–2.2 cm, green to vinaceous to purple, with a mixture of sparse eglandular (scabrid) and sparse or more numerous glandular, hyaline hairs, cincinnus internodes 0.2–1.1 cm long, green to vinaceous to purple, with a mixture of sparse eglandular (scabrid) and sparse or more numerous glandular, hyaline hairs; bracteoles ca. 1.8–3.7 × 0.9–1 mm, persistent, triangular to broadly triangular, cup-shaped, light green to lilac, glabrous to sparsely pilose, base amplexicaul, non-perfoliate, margins glabrous or rarely sparsely ciliate, apex acuminate. *Flowers* bisexual or male, enantiostylous, ca. 0.5–1.2 cm diameter; floral buds narrowly ovoid to ovoid, 2.1–4 × 1–2 mm, green to lilac; pedicels 0.3–1 cm long, green to vinaceous to purple, with a mixture of sparse eglandular (scabrid) and sparse or more numerous glandular, hyaline hairs, erect and elongate in fruit; sepals 3.2–5 × 1.5–2 mm, triangular to ovate-triangular, cucullate, green, glandular to densely glandular, hyaline hairs, apex acuminate, margins hyaline light green to hyaline lilac; petals equal, 4–6.3 × 3–4.2 mm, obovate to narrowly obovate, slightly cucullate, pale lilac to lilac to pink, rarely white, glabrous, base cuneate, margins entire, apex obtuse to rounded; stamens 3, equal, filaments glabrous, gently curved at the apex, 3.8–5.2 mm long, pale lilac to lilac or white, anthers narrowly elliptic to narrowly oblong, 0.8–1.0 × 0.3–0.7 mm, connective lilac, anthers sacs white, pollen white; staminodes 3, equal, filaments glabrous, straight, 1.6–2.1 mm long, pale lilac to white, antherodes sagittate, 0.8–0.9 × 0.9–1.0 mm, connective golden yellow, lobes conspicuous, cream-colored to pale yellow; ovary ellipsoid to oblongoid, 0.9–1.8 × 0.6–0.8 mm, 3-locular, white to light green, smooth, glabrous, style gently curved at the apex, ca. 1.8–3.6 mm, pale lilac to lilac or white, stigma truncate, white to lilac. *Capsules* 2.8–4.4 × 3–4.8 mm, subglobose to globose, apiculate due to persistent style, 3-locular, 3-valved, light brown when mature, glabrous, smooth. *Seeds* 1 per locule, 1.9–2.8 × 1.3–2.1 mm, reniform to broadly ellipsoid, cleft towards the embryotega, ventrally flattened, testa dark brown to greyish brown, densely farinose, costate to slightly rugose, with ridges radiating from the embryotega, with a tan appendage that extends ventri-laterally to the embryotega and basally into the hilum; embryotega semilateral, relatively inconspicuous, generally covered by a cream farina, without a prominent apicule; hilum linear, approximately the same length as the seed, in a deep depression.

#### Specimens seen.

**BOLIVIA. Santa Cruz**: San Ignacio de Velasco, 30 km acia S, 12 Apr 1988, B. Bruderreck 310 (LPB, US). **BRAZIL. Goiás**: Provincia Goyaz, Salinas, May–Jul 1844, M.A. Weddell 2103 (P); loc. cit., May–Jul 1844, M.A. Weddell 2106 (P); s.loc., 1841, G. Gardner 3481 (K); Colinas do Sul, Vila Borba, 15 Jun 1993, G. Hatschbach et al. 59587 (MBM, MO, USU); Formoso, arredores de Formoso, 3 May 2012, R.J.V. Alves 8898 (R); Paraíso, ca. 27 km sul de Paraíso, 23 Mar 1968, H.S. Irwin et al. 21659 (K, NY, UB); loc. cit., 23 Mar 1968, H.S. Irwin et al. 21717 (NY, UB); **Maranhão**: Carolina, 1 Jun 1950, J.M. Pires & G.A. Black 2564 (UFMA, US); **Pará**: Ilha do Marajó, 15 Aug 1901, fl, fr., M. Guedes 2314 (BM); Serra do Cachimbo, Jun 1955, M. Alvarenga s.n. (RB 90541); loc. cit., 12 Dec 1956, J.M. Pires et al. 6104 (NY, UFMA); Itaituba, arredores da base aérea do Cachimbo, 25 Apr 1983, M.N. Silva et al. 90 (INPA, K, MG, NY, US); loc. cit., 26 Apr 1983, M.N. Silva et al. 118 (INPA, MG, NY, US); **Piauí**: Piauhy, Parnaguá, marshy places, Aug–Sep 1839, fl., fr., G. Gardner 2743 (BM, K); **Tocantins**: Araguaina, 20 km ao Sul, 26 Mar 1976, G. Hatschbach & R. Kummrow 38378 (MBM, US). **VENEZUELA. Apure**: Departamento Muñoz, módulos F. Corrales de la UNELLEZ, entre los caños Guaritico y Caicara, 25 Oct 1980, B. Stergios 2379 (PORT, US); loc. cit., 10 Sep 1981, fr., G. Aymard 466 (PORT); loc. cit., 13 Sep 1981, B. Stergios et al. 9568 (PORT, US); loc. cit., 9 Dec 1986, G. Aymard & R. Schargel 5017 (PORT, US); loc. cit., 12 Dec 1986, G. Aymard & R. Schargel 5071 (PORT, US); **Cojedes**: San Carlos, en extremo Sur del Hato “El Laurel”, mas o menos km. 17 al sur de San Carlos, 21 Aug 1976, fl., fr., B. Trujillo 13843 (MY); **Guárico**: Calabozo, ca. 39 km SSW of Calabozo on Hato Masaguaral, 17 Sep 1983, R. Rondeau 469 (US); **Portuguesa**: Guanare, terrenos de la UNELLEZ, Mesa de Cavacas, 6 Sep 1986, fl., fr., B. Stergios 7151 (PORT).

#### Distribution and habitat.

**Figure 10.**
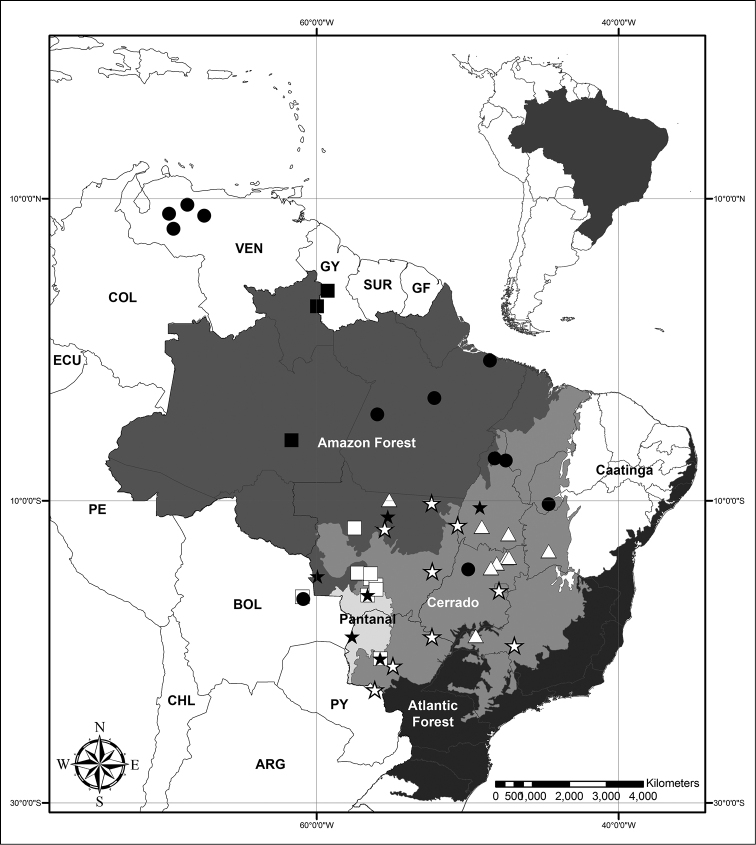
Distribution map of Neotropical *Murdannia* Royle. **Full circles** *Murdannia burchellii* **Full stars** *Murdannia engelsii* **Triangles** *Murdannia gardneri* **Stars** *Murdannia paraguayensis* **Full squares** *Murdannia schomburgkiana* **Squares** *Murdannia semifoliata*.

*Murdannia burchellii* has a very fragmented distribution, probably due to lack of collections, being known to occur in Bolivia, Brazil (in the states of Goiás, Maranhão, Pará, Piauí and Tocantins), and Venezuela (Fig. 10). It grows in shady to open sandy river banks of the Amazon and Cerrado domains.

#### Phenology.

It was found in bloom and fruit from October to July.

#### Conservation status.

*Murdannia burchellii* possesses a wide EOO (ca. 3,513,319.273 km^2^), but due to the few and scattered collections known for this species, its AOO is considerably smaller (ca. 22,500.000 km^2^). Thus, following the IUCN recommendations (IUCN 2001), *Murdannia burchellii* should be considered Least Concern. Nonetheless, it is important to highlight the small number of collections and how fragmented the distribution of *Murdannia burchellii* is. Also, the most recent collection was made in 1993. Which may indicate an ongoing decrease of size of the subpopulations and the loss of habitat for this species.

#### Nomenclatural notes.

When describing *Aneilema gardneri* var. *burchelli*, Clarke (1881) lists two collections (*W.J. Burchell 8165* and *M.A. Weddell 2106*). Since the name of Clarke’s new variety honors W.J. Burchell, it seems logical to designate his collection as the lectotype. Aside from that, this collection is well distributed in several herbaria around the world. Furthermore, the specimen from K herbarium matches Clarke’s description and has hand annotations made personally by Clarke. Thus, we designate this specimen as the lectotype for *Aneilema gardneri* var. *burchelli*.

When describing Aneilema gardneri var. glabrior, Clarke (1881) cites three collections by *G. Gardner* (2743, 3481, 4020). The specimen of at K *Gardner 4020* is mounted on the same sheet as *Gardner 3481*, and both being annotated by Clarke as Aneilema gardneri var. glabrior. *Gardner 4020* is also the most well distributed of the three collections. Nonetheless, the specimen of at K represents *Murdannia gardneri*, so it cannot be designated as the type of Aneilema gardneri var. glabrior. Thus, the specimen at K is not considered part of the original material. One of us (RBF) examined and recorded a specimen of *Gardner 4020* at BM in 1993, with the following data on the label: “Moist campos between Natividade and Conceição, Feby 1840, Herb. Gardner.” While this would appear to be the most logical choice for a lectotype, the specimen was not photographed when other types were photographed at BM, and it cannot be found today. Therefore, the specimen at P is here designated as the lectotype. This specimen also bears an identification in Clarke’s handwriting.

#### Discussion.

*Murdannia burchellii* is morphologically similar to *Murdannia gardneri* due to the general aspect of the plants, indumentum and by the presence of a ventri-lateral appendage in the seeds. It was traditionally treated as part of *Murdannia gardneri* *s.l.* due to the number of cincinni per inflorescence, the posture of the pedicels at post-anthesis and in fruit, general floral and capsule morphology, and due to the hilum being positioned in a deep depression (Table 1). Nevertheless, both species can be readily differentiated by the stature and robustness of the plants, the insertion of the cincinni in the main axis of the inflorescence and testa ornamentation. Furthermore, the cincinni in *Murdannia burchellii* are conspicuously sinuate, while the cincinni in *Murdannia gardneri* are straight. After analyzing the syntypes for Aneilema gardneri var. glabrior, it became clear that they were conspecific with *Murdannia burchellii*. All specimens possess the characteristic alternate to subopposite cincinni, being differentiated only from *Murdannia burchellii* by sparser eglandular and glandular hairs in the inflorescence. All the analyzed specimens possessed some type of indumentum in the inflorescence, despite Clarke’s description (1881) stating they were completely glabrous.

Some young specimens of *Murdannia burchellii* with inflorescences reduced to a solitary cincinnus, can be confused with specimens of *Murdannia engelsii*. Nevertheless, these can be differentiated by their glabrous stems, leaf-blades with truncate base, sinuate cincinni, cup-shaped bracteoles, and glabrous androecium and gynoecium (vs. stems with glandular hairs, leaf-blades with an amplexicaul base, straight cincinni, flat bracteoles and minutely glandular-pubescent androecium and gynoecium in *Murdannia engelsii*) (Table 1).

### 2. Murdannia engelsii

Taxon classificationPlantaeCommelinalesCommelinaceae

M.Pell. & Faden sp. nov.

urn:lsid:ipni.org:names:77158529-1

Figs 2 , 10

#### Diagnosis.

Similar to *Murdannia paraguayensis* due to its deflexed pedicels at post-anthesis and when fruiting; petals with minute glandular hairs at base on the adaxial surface; filaments, ovaries, styles and capsules with minute glandular hairs, and capitate stigma. It can be differentiated by its trailing stems, distichously-alternate leaves, inflorescence reduced to a solitary cincinnus, peduncles with a mixture of eglandular and glandular hairs, cincinni 2–7-flowered, capsules broadly ovoid to broadly ellipsoid, and 1-seeded locules.

#### Type.

BRAZIL. Mato Grosso: Itaúba, Resgate de Flora da UHE Colíder, lote G de supressão, 260 m, floresta do Planalto dos Parecís, prainha arenosa no rio Teles Pires, fl., fr., 27 May 2015, *M.E. Engels et al. 3474* (holotype: RB!; isotypes: CNMT!, HERBAM!, MBM!, US!, TANG!).

#### Description.

*Herbs* ca. 10.0–36.0 cm tall, perennial, rhizomatous without a definite base, terrestrial to paludal in river banks. *Roots* thin, fibrous, brown, densely to sparsely pilose with hyaline hairs, emerging from the basalmost nodes and rhizome. *Rhizomes* long, trailing, light brown to light green, shallowly buried in the sand. *Stems* ascending to erect, thin, herbaceous to slightly succulent, usually densely branched or branched only at the base, sometimes branching from the upper nodes; internodes 1.3–3.5 cm long, green, with a mixture of eglandular (scabrid) and glandular hairs, becoming glabrous with age, with a line of eglandular hairs opposite the leaf above, hairs hyaline. *Leaves* distichously-alternate, evenly distributed along the stems, rarely somewhat congested at the apex of the stems, the distal ones gradually smaller than the proximal ones; sheaths 2–2.5 mm long, green, with glandular hairs, becoming glabrous with age, hairs hyaline, margins sparsely ciliate, with a line of eglandular hairs opposite the leaf above, hairs hyaline; lamina (0.5–)1.6–6 × 0.3–1 cm, membranous, generally conduplicate, rarely flat, slightly falcate to falcate, green on both sides, drying olive-green on both sides, narrowly elliptic to narrowly lanceolate or narrowly ovate, glabrous on both sides or the uppermost usually with glandular hairs at least basally, base amplexicaul, margins green, ciliate to setose at base or the uppermost sometimes with glandular hairs, apex acuminate; midvein slightly conspicuous, slightly impressed adaxially, prominently acute abaxially, secondary veins 2(–3) pairs, inconspicuous to slightly conspicuous on both sides, dark green. *Inflorescences* 1–2–(5), terminal or axillary from the uppermost nodes, consisting of a solitary cincinnus; peduncles 1–1.4 cm, with a mixture of eglandular (scabrid) and glandular to densely glandular hyaline hairs; basal bract reduced, 5–5.5 × 4–4.5 mm, lanceolate to ovate, adaxially glabrous, abaxially glabrous or with glandular hairs, base amplexicaul, margins ciliate at base, apex acute, veins inconspicuous on both sides, dark green; cincinni 2–7-flowered, erect, straight, peduncle 3.5–8 mm long, green, with glandular to densely glandular, hyaline hairs, cincinnus internodes 4.5–8 mm long, green, with glandular to densely glandular hyaline hairs; cincinnus bract and bracteoles ca. 1–1.5 × 0.9–1 mm, persistent, ovate, flat, light green, with a sparse mixture of eglandular (scabrid) and glandular hairs near the base, base amplexicaul, non-perfoliate, margins glabrous, apex acute. *Flowers* bisexual or male, enantiostylous, 1–1.4 cm diam.; floral buds ovoid, 2.8–3.1 × 2.5–3 mm, green; pedicels 1–6 mm long, green, with glandular to densely glandular, hyaline hairs, deflexed and slightly elongate in fruit; sepals 3–3.5 × 0.5–0.8 mm, triangular to ovate-triangular, cucullate, green, with glandular to densely glandular, hyaline hairs, apex acute, margins hyaline light green; petals equal, 4.5–7.3 × 2.5–4.5 mm, obtrullate, rarely obovate, slightly cucullate, pale lilac to lilac, mauve or pink, rarely white, with minute glandular hairs at the base on the adaxial surface, base cuneate, margins entire, apex obtuse to rounded; stamens 3, equal, filaments basally with minute glandular hyaline hairs, gently curved in the middle, 4.1–5.9 mm long, pale lilac to lilac or white, anthers elliptic, 0.6–0.7 × 0.3–0.7 mm, connective white to lilac, anthers sacs white to pale lilac, pollen white; staminodes 3, equal, filaments with minute glandular hyaline hairs, straight, 1.3–1.7 mm long, white to pale lilac, antherodes subsagittate to subcordate, 0.9–1.0 × 0.9–1.0 mm, connective golden yellow, lobes conspicuous, cream-colored to pale yellow; ovary ovoid to ellipsoid, 0.9× 0.7–0.8 mm, 3-locular, white to light green, smooth, with minute glandular hyaline hairs, style curved at the apex, ca. 3.6–8 mm, white to pale lilac or lilac, stigma capitate, white to lilac. *Capsules* 3-locular, 3-valved, 3.2–4.5 × 2–2.5 mm, broadly ovoid to broadly ellipsoid, apiculate due to persistent style, light brown when mature, with minute glandular hyaline hairs, sometimes glabrescent with age, smooth. *Seeds* 1 per locule, 1.8–2.0 × 1–1.2 mm, reniform to broadly ellipsoid, cleft towards the embryotega, ventrally flattened, testa medium to dark brown, sparsely farinose, scrobiculate to shallowly scrobiculate, with ridges radiating from the embryotega, sometimes with 4–7 ventral furrows, with a tan appendage that extends ventri-laterally to the embryotega and basally into the hilum; embryotega semilateral to semidorsal, relatively inconspicuous, generally covered by a cream farina, without a prominent apicule; hilum linear, approximately the same length as the seed, in a shallow depression.

#### Specimens seen

**(paratypes). BRAZIL. Mato Grosso**: Itaúba, resgate de Flora da UHE Colíder, Lote G de supressão, floresta do Planalto dos Parecís, região de ecótono entre a Floresta Amazônica e Cerrado, 3 Jun 2016, M.E. Engels & A.S. Bezerra 4510 (HERBAM, MBM, RB); Poconé, rodovia Transpantaneira, 17 May 1983, J. Barcia et al. 1560 (R); loc. cit., Fazenda Nova Berlim, Transpantaneira highway, km 85, 3 May 1992, M. Schessl 2602b (CH, UFMT, ULM, US); loc. cit., highway Poconé-Porto Cercado, 30 May 1992, M. Schessl 2631g (CH, CPAP, UFMT, ULM, US); loc. cit., estrada para Porto Cercado, km 18, 22 Apr 1993, A.L. Prado 2017 (UEC, UFMT); loc. cit., Fazenda Ipiranga, 8 May 1993, A.L. Prado & R. Ribeiro 2045 (HURB, UEC, UFMT); loc. cit., Fazenda Ipiranga, Pousada Piuvial, vazante da sede, km 11 da rodovia Transpantaneira, 20 May 1996, V.J. Pott et al. 3186 (CPAP, US); Vila Bela da Santíssima Trindade, Parque Estadual Serra de Ricardo Franco, margem do rio Guaporé, 23 May 1978, P.G. Windisch 1863 (RB); **Mato Grosso do Sul**: Corumbá, Fazenda Caceres, próximo da sede de Nhecolândia, 12 Aug 1988, V.J. Pott et al. 595 (CPAP, MBM, US); loc. cit., Fazenda Alegria, Nhecolândia, 30 Jul 1989, A. Pott et al. 4912 (CPAP, MBM, US); loc. cit., próximo ao mata burro na divisa com Retiro Mandovi, Nhecolândia, 3 Aug 1999, V.J. Pott & A. Rodrigues 3993 (CPAP, US); **Tocantins**: Pium, Ilha do Bananal, Parque Nacional do Araguaia, base física do rio Javaés, antigo acampamento do Projeto Quelônios do Amazônia, 27 Mar 1999, M. Aparecida da Silva et al. 4167 (IBGE, RB).

#### Etymology.

The epithet honors the collector of the holotype, the Brazilian botanist Mathias Erich Engels, Orchidaceae taxonomist and dear friend of the authors.

#### Distribution and habitat.

*Murdannia engelsii* is endemic to Brazil, being known from the states of Tocantins, Mato Grosso and Mato Grosso do Sul (Fig. 10). It grows in shady to open sandy river banks of the Amazon, Cerrado and Pantanal domains. Its prostrate stems produce dense mats, generally near rocks and grasses.

#### Phenology.

It was found in bloom and fruit from March to August.

#### Conservation status.

*Murdannia engelsii* possesses both a wide EOO (ca. 514,893.048 km^2^) and a wide AOO (ca. 15,000.000 km^2^). Following the IUCN recommendations (IUCN 2001), *Murdannia englesii* should be considered Least Concern. Nevertheless, most of the known populations of *Murdannia engelsii* are in areas currently being deforested and turned into pasture sites for cattle. We believe that this species is highly affected by human activity and should be considered Vulnerable [VU, A2cd+ B2ab(ii, iii,v)+D2].

#### Discussion.

*Murdannia engelsii* is morphologically similar to *Murdannia burchellii*, *Murdannia gardneri* and *Murdannia paraguayensis* due to indumentum and flower morphology, and also similar to *Murdannia paraguayensis* due to the deflexed pedicels in fruit. However, *Murdannia engelsii* can be easily differentiated by its inflorescence reduced to a solitary cincinnus (*vs.* thyrsi with several, verticillate or alternate to subopposite cincinni). It can be easily differentiated from *Murdannia burchellii* and *Murdannia gardneri* by inflorescence morphology, position of the pedicels at post-anthesis and in fruit, by the indumentum of the filaments, gynoecium and capsules, and seed morphology. *Murdannia engelsii* is much more similar to *Murdannia paraguayensis*, due to several key characters. These are the only species in the genus to have petals with minute glandular hairs at the base on the adaxial surface, androecium and gynoecium with glandular hairs, and the only Neotropical species to have pedicels deflexed post-anthesis and in fruit. Nevertheless, *Murdannia engelsii* can be differentiated by its trailing habit (*vs.* erect in *Murdannia paraguayensis*), leaves distichously-alternate (*vs.* spirally-alternate), inflorescence reduced to a solitary cincinnus (*vs.* inflorescence with several verticillate cincinni), cincinni 2–7-flowered (*vs.* 1-flowered), capsules broadly ovoid to broadly ellipsoid (*vs.* oblongoid to broadly oblongoid), and locules 1-seeded (*vs.* 2-seeded). *Murdannia engelsii* can also be confused with *Murdannia nudiflora*, due to their small stature, phyllotaxy and inflorescence morphology. However, they can be easily differentiated by its erect cincinni (*vs.* pendulous), persistent bracteoles (*vs.* caducous), corolla actinomorphic (*vs.* zygomorphic), three stamens and three staminodes (*vs.* two stamens and four staminodes), filaments with minute glandular hairs (*vs.* bearded with moniliform hairs), and locules 1-seeded (*vs.* locules 2-seeded) (Table 1). One of the most striking features of *Murdannia engelsii* would be occasional production of several inflorescences clustered towards the apex of a shoot, forming a synflorescence. This synflorescence resembles a single inflorescence with several alternate cincinni.

### 3. Murdannia gardneri

Taxon classificationPlantaeCommelinalesCommelinaceae

(Seub.) G.Brückn., Nat. Pflanzenfam. (ed. 2)15a: 173. 1930.

Figs 3 , 4 , 10

Phaeneilema gardneri (Seub.) G.Brückn., Notizbl. Bot. Gart. Berlin–Dahlem 10 (91): 56. 1927.
Aneilema gardneri Seub., in Martius, Fl. Bras. 3 (1): 259. 1855. Lectotype (designated here): BRAZIL. Goyaz, moist places near Villa de Arrayal, fl., fr., April 1841, G. Gardner 4021 (K barcode K000363236!; isolectotypes: B barcode B100367834!, BM barcodes BM001172132!, BM001172133!, G barcodes G00098261!, G00098262!, G00165012!, K barcode K000363237!, NY barcodes NY00247400!, NY00247401!, P barcode P02088022!, US barcode US00091574!).

#### Description.

*Herbs* ca. 30.0–150.0 cm tall, perennial, rhizomatous with a definite base, terrestrial to paludal to rooted emergent in flooded fields. *Roots* thin, fibrous, medium to dark brown, densely to sparsely pilose with medium to dark brown hairs, emerging from the short rhizome and from the basalmost nodes. *Rhizomes* short, light to medium brown, buried in the sand or ground. *Stems* prostrate, with erect to ascending apex, succulent, unbranched to little-branched at the base; internodes 1.9–10.7 cm long, green to vinaceous, glabrous to sparsely pilose or hispid, becoming glabrous with age, with a line of eglandular hairs opposite the leaf above, hairs hyaline. *Leaves* spirally-alternate, evenly distributed along the stems, sessile, the distal ones gradually reduced; sheaths 0.5–3.2 cm long, green to vinaceous, sparsely pilose to hispid, becoming glabrous with age, hairs hyaline, margins ciliate to hispid, with a line of eglandular hairs opposite the leaf above, hairs hyaline; lamina 4.2–17.4 × 0.7–1.3 cm, chartaceous, conduplicate, slightly falcate to falcate, green on both sides, drying light brown to olive-green on both sides, linear-lanceolate to lanceolate, sparsely pilose to hispid, becoming glabrous with age, rarely glabrous, base truncate to rounded, margins light green, ciliate to setose only at base, apex acuminate; midvein inconspicuous, slightly impressed adaxially, slightly obtuse abaxially, secondary veins 3–4(–5) pairs, adaxially inconspicuous to slightly conspicuous, light green, abaxially somewhat conspicuous. *Inflorescences* 1–(3) thyrsi, terminal or axillary from the uppermost nodes, thyrse with 16–38 verticillate cincinni, arranged in 2–9 whorls; peduncles 2.7–8.4 cm, with a mixture of eglandular (scabrid) and glandular, hyaline hairs; basal bract leaf-like, 2.4–7.2 × 0.3–0.9 cm, linear-lanceolate to lanceolate, sparsely pilose to hispid, rarely glabrous, base rounded, margins ciliate to setose only at base, apex acuminate, veins inconspicuous, concolorous to light green; cincinni bracts ca. 0.4–0.8 × 0.1–0.3 cm, ovate to broadly ovate, cup-shaped, light green to lilac, glabrous to pilose, base truncate, margins glabrous to sparsely ciliate, apex acuminate; cincinni 2–11-flowered, ascending, straight, peduncle 0.5–1.3 cm, light green to vinaceous to purple, with a mixture of eglandular (scabrid) and glandular or all glandular hyaline hairs, internodes 0.9–5.2 mm long, light green to vinaceous to purple, with a mixture of eglandular (scabrid) and glandular or all glandular, hyaline hairs; bracteoles ca. 1.8–4.1 × 2.8–4.2 mm, persistent, broadly ovate to depressed ovate, cup-shaped, light green to lilac or pink, sparsely pilose, base amplexicaul, non-perfoliate, margins glabrous to ciliate, apex acuminate. *Flowers* bisexual or male, enantiostylous, ca. 1.4–2.3 cm diam.; floral buds narrowly ovoid to ovoid, 2.6–5.3 × 1.2–2.4 mm, light green to pink to vinaceous; pedicels 2.2–7.3 mm long, light green to vinaceous to purple, with a mixture of eglandular (scabrid) and glandular or all glandular, hyaline hairs, erect and elongate in fruit; sepals 3.6–6.1 × 3.2–4.8 mm, triangular to ovate-triangular, cucullate, green to lilac to vinaceous to purple, with glandular to densely glandular, hyaline hairs, apex acuminate, margins hyaline light green to hyaline pink; petals equal, 0.7–1.2 × 0.6–0.8 cm, obovate to elliptic-obovate, slightly cucullate, pale lilac to lilac, purple or pink, rarely white, glabrous, base cuneate, margins entire, apex acute to obtuse; stamens 3, equal, filaments glabrous, gently curved at the apex, 6.2–9.4 mm long, pale lilac to lilac or white, anthers elliptic, 0.7–0.9 × 0.3–0.4 mm, connective lilac to white, anthers sacs white to lilac, pollen white; staminodes 3, equal, filaments glabrous, straight, 3.1–5.3 mm long, pale lilac to white, antherodes cordate, 0.7–0.9 × 0.8–0.9 mm, connective golden yellow, lobes conspicuous, cream-colored to pale yellow; ovary ellipsoid to broadly ellipsoid, 0.6–0.8 × 0.4–0.6 mm, 3-locular, white to light green, smooth, glabrous, style gently curved at the apex, ca. 4.8–6.2 mm, pale lilac to lilac or white, stigma truncate, white to lilac. *Capsules* 3.6–4.5 × 3.4–4.2 mm, 3-locular, 3-valved, subglobose to globose, apiculate due to persistent style, light brown when mature, glabrous, smooth. *Seeds* 1 per locule, 1.9–2.6 × 1.2–1.8 mm, reniform to broadly ellipsoid, strongly cleft towards the embryotega, ventrally flattened, testa dark brown to greyish brown, sparsely farinose, scrobiculate to foveolate, with ridges radiating from the embryotega, with a tan appendage that extends ventri-laterally to the embryotega and basally into the hilum; embryotega semilateral, relatively inconspicuous, without a prominent apicule; hilum linear, approximately the same length as the seed, in a deep depression.

#### Specimens seen.

**BRAZIL. Bahia**: Correntina, Chapadão Ocidental da Bahia, Islets and banks of the rio Corrente, 23 Apr 1980, R.M. Harley et al. 21668 (CEPEC, HRB, K, MBM, US); loc. cit., 21 Jan 1997, G. Hatschbach et al. 66044 (MBM); **Goiás**: Cavalcante, estrada rio Trairas/rio Custódio, km 2, 28 Nov 2006, G. Pereira-Silva & G.A. Moreira 11159 (CEN, RB); Colinas do Sul, fazenda Saracura, estrada de manutenção das novas linhas de transmissão Minacu/Niquelândia, 8 Sep 1995, B.M.T. Walter et al. 2604 (CEN, RB); Goyaz, 1841, G. Gardner 4020 (K barcode K000363238, US barcode US00160560); Itumbiara, rodovia Itumbiara–Rio Verde, a 31 km de Itumbiara, 18 Apr 1978, G.J. Shepherd et al. 7415 (F ex UEC); Niquelândia, 27 km de Colinas em direção a Niquelândia, próximo ao rio Tocantinzinho, 6 May 1998, M.A. Silva et al. 3772 (IBGE, RB, US); Teresina de Goiás, km 12 da estrada GO-118, sentido Nova Roma, 29 April 1996, B.A.S. Pereira & D. Alvarenga 3027 (IBGE, RB); **Mato Grosso**: Novo Mundo, Parque Estadual do Cristalino, entrada para Fazenda J.J., 26 January 2008, D. Sasaki et al. 1934 (HERBAM, HURB, SPF); **Minas Gerais**: Ituiutaba, 26 May 1946, A. Macedo 760 (US); **Tocantins**: Conceição do Tocantins, rodovia TO-050, km 375, fazenda São José, próximo do rio Santa Isabel, 11 May 2000, G. Hatschbach et al. 70903 (MBM); Gurupi, próximo à Gurupi, 20 Apr 1978, R.P. Orlandi 73 (HRB, RB).

#### Distribution and habitat.

*Murdannia gardneri* is endemic to Brazil, being known from the states of Bahia, Goiás, Mato Grosso, Minas Gerais and Tocantins (Fig. 10). It grows in open sandy river banks or flooded grass fields, of the Cerrado domain.

#### Phenology.

It was found in bloom and fruit throughout the year.

#### Conservation status.

*Murdannia gardneri* possesses a EOO of ca. 497,658.992 km^2^ and a AOO of ca. 20,000.000 km^2^. Most of the known collections are concentrated in central Brazil, where the native vegetation is commonly removed to give place to livestock. This is especially common in the Cerrado domain, due to its savanna vegetation being easier to remove than the dense rainforests of the Amazon and Atlantic Forest domains. Thus, we believe that *Murdannia gardneri* should be considered Nearly Threatened.

#### Nomenclatural notes.

When describing *Aneilema gardneri*, Seubert (1855) lists two collections from G. Gardner (*4020* and *4021*). As aforementioned, *Gardner 4020* consists of a mixture of *Murdannia burchellii* and *Murdannia gardneri*. Fortunately, the same is not true for *Gardner 4021*. Furthermore, the *Gardner 4020* specimen at P was designated by us as the lectotype for Aneilema gardnei var. glabrior. Thus, we designate a specimen at K as the lectotype for *Aneilema gardneri*.

#### Discussion.

*Murdannia gardneri* is morphologically similar to *Murdannia burchellii* and *Murdannia paraguayensis* due to their phyllotaxy and by the number of cincinni per inflorescence. It is morphologically more similar to *Murdannia burchellii* due to the posture of the pedicels at post-anthesis and when fruiting, general floral and capsule morphology, and due to the hilum being positioned in a deep depression. Nevertheless, both species can be easily differentiated based on the insertion of the cincinni in the main axis of the inflorescence (alternate to subopposite in *Murdannia burchellii* vs. verticillate in *Murdannia gardneri*), the ornamentation of the testa (costate to slightly rugose *vs.* scrobiculate to foveolate), robustness of the plants (delicate *vs.* robust, branching pattern (densely branched at base *vs.* unbranched to little-branched), leaf-blade consistency (chartaceous *vs.* succulent), and some indumentum differences. On the other hand, *Murdannia paraguayensis* can be readily differentiated from *Murdannia gardneri* by its 1-flowered cincinni (*vs.* many-flowered in *Murdannia gardneri*), deflexed pedicels post-anthesis and when fruiting (*vs.* erect), filaments with minute glandular hairs (*vs.* glabrous), gynoecium and capsules with glandular hairs (*vs.* glabrous), capsule oblongoid to broadly oblongoid (*vs.* subglobose to globose), locules 2-seeded (*vs.* 1-seeded), and hilum in a shallow depression (vs. in a deep depression) (Table 1).

### 4. Murdannia nudiflora

Taxon classificationPlantaeCommelinalesCommelinaceae

(L.) Brenan, Kew Bull. 7(2): 189. 1952.

Fig. 5

Phaeneilema nudiflorum (L.) G.Brückn., Notizbl. Bot. Gart. Berlin–Dahlem 10 (91): 56. 1927.
Ditelesia nudiflora (L.) Raf., Fl. Tellur. 3: 69. 1837.
Aneilema nudiflorum (L.) R.Br., Prodromus Florae Novae Hollandiae: 271. 1810.
Commelina nudiflora L., Sp. Pl. 1: 41. 1753. Lectotype (designated by Merrill 1937): INDIA. s.loc., fl., fr., s.dat., P. Osbeck s.n. (LINN barcode LINN-HL65-12!).

#### Diagnosis.

*Herbs* annual, with a definite base, terrestrial to paludal to rooted emergent in flooded fields. *Roots* thin, fibrous, brown, densely to sparsely pilose, emerging from the basal most nodes. *Rhizomes* absent. *Stems* prostrate, erect to ascending apex, unbranched or branched at the base, glabrous. *Leaves* distichously-alternate, distributed along the stems, rarely 1–2 congested at base, the distal ones gradually smaller than the basal ones; lamina membranous, conduplicate, linear to linear-lanceolate or lanceolate-oblong, glabrous or with eglandular hairs. *Inflorescences* 1–(2), terminal or axillary from the uppermost node, long-pedunculate, exerted from the leaf-sheaths, consisting of a solitary cincinnus; basal bract inconspicuous; cincinni bracts cup-shaped; cincinni 2–12-flowered, pendent, bracteoles cup-shaped, caducous. *Flowers* bisexual or male, zygomorphic due to the position of the lateral petals; pedicels erect and elongate in fruit; sepals ovate-elliptic to ovate-triangular, cucullate, glabrous; petals subequal, obovate to spatulate to obtrullate, slightly cucullate, pale lilac to purple or mauve, glabrous; stamens 2 (opposite to the lower petals), equal, filaments gently sigmoid, closely parallel to each other, white at the base, lilac at the middle, purple at the apex, densely bearded with moniliform, purple hairs, anthers elliptic to oblong, connective bluish lilac to white, anthers sacs purple to dark purple, pollen white; staminodes 4, 1 staminode antesepalous, opposite to the lower sepal, filament white to lilac, medially bearded with moniliform, purple hairs, antherode small, white, sometimes lacking, 3 antepetalous, filaments straight, pale lilac to white, glabrous or sparsely medially bearded with moniliform, purple hairs, antherodes hastate, white to cream; ovary ellipsoid to oblongoid, 3-locular, light green smooth, glabrous, style strongly curved at the apex, white to pale lilac, glabrous, stigma capitate, lilac. *Capsules* 3-locular, 3-valved, ovoid to subglobose, apiculate due to persistent style, light brown when mature, smooth, glabrous. *Seeds* 2 per locule, broadly ellipsoid to oblongoid, not cleft towards the embryotega, ventrally ridged, testa yellowish brown to brown, foveolate-reticulate, with pale warts around depressions, farinose, appendage absent; embryotega semilateral, relatively inconspicuous, without a prominent apicule; hilum elliptic, approximately ½ the length of the seed, on a weak ridge.

#### Distribution and habitat.

Native to Tropical Asia to Malaysia and naturalized in West Africa, North America, Central America, the West Indies and South America; in the New World ranging from the southeastern United States to Argentina. In Brazil it is known to occur in the states of Acre, Alagoas, Amazonas, Bahia, Ceará, Goiás, Maranhão, Mato Grosso, Mato Grosso do Sul, Minas Gerais, Pará, Paraíba, Paraná, Rio Grande do Sul, Santa Catarina, São Paulo and Tocantins, in disturbed vegetation, roadsides and near rice crops.

#### Phenology.

It was found in bloom and fruit throughout the year.

#### Discussion.

*Murdannia nudiflora* can be easily recognized by its caduceus bracteoles, single terminal cincinni, two fertile stamens and four staminodes, and capsules with 2-seeded locules (Table 1).

### 5. Murdannia paraguayensis

Taxon classificationPlantaeCommelinalesCommelinaceae

(C.B.Clarke ex Chodat) G.Brückn., Nat. Pflanzenfam. (ed. 2)15a: 173. 1930.

Figs 6 , 10

Phaeneilema paraguayensis (C.B.Clarke ex Chodat) G.Brückn., Notizbl. Bot. Gart. Berlin–Dahlem 10 (91): 56. 1927.
Aneilema paraguayense C.B.Clarke ex Chodat, Bull. Herb. Boissier, sér. 2, 1: 437. 1901. Lectotype (designated here): PARAGUAY. Departamento de Canindeyú: Sierra de Maracayú, fl., fr., Oct 1898–1899, E. Hassler 5083 (G barcode G00195432!; isolectotypes: BM barcode BM000526690!; G barcode G00009034!, NY barcode NY00247403!).

#### Description.

*Herbs* ca. 20.0–150.0 cm tall, perennial, rhizomatous with a definite base, terrestrial to paludal to rooted emergent in flooded fields. *Roots* thin, rarely thick, fibrous, medium to dark brown, densely to sparsely pilose with medium to dark brown hairs, emerging from the short rhizome and from the basalmost nodes. *Rhizomes* short, light to medium brown, buried in the sand or ground. *Stems* prostrate, with erect to ascending apex, succulent, unbranched or only branched at the base; internodes 3.4–13.0 cm long, green to vinaceous, glabrous to sparsely pilose, becoming glabrous with age, with a line of eglandular hyaline hairs opposite the leaf above. *Leaves* spirally-alternate, sometimes becoming distichously-alternate at apex, evenly distributed along the stems, the distal ones gradually smaller than the basal ones; sheaths 1.2–3.3 cm long, green to vinaceous, glabrous to pilose along the fused edge, sometimes with a few scattered long, glandular hairs, margins ciliate to sparsely setose at base, hairs hyaline; lamina 2.5–23.6 × 0.4–1.2 cm, succulent, canaliculate, slightly falcate to falcate, green on both sides, drying light brown to olive-green or light green on both sides, linear-lanceolate to linear-elliptic or linear-oblong, glabrous, base truncate to rounded, margins light green to pink or vinaceous, ciliate to sparsely setose only at base, apex acute to acuminate; midvein conspicuous or inconspicuous, slightly impressed adaxially, slightly obtuse abaxially, secondary veins 2–3(–4) pairs, adaxially inconspicuous or slightly conspicuous, light green, abaxially slightly conspicuous. *Inflorescences* 1–(2), thyrsi, terminal or axillary from the uppermost node, thyrse with 9–24 verticillate cincinni, arranged in 3–9 whorls; peduncles 1.2–7.5 cm, with glandular to densely glandular, hyaline hairs; basal bract leaf-like, 2.1–3.2 × 0.9–1.2 cm, lanceolate, glabrous, base rounded, margins ciliate to setose only at base, apex acute to acuminate, veins inconspicuous or slightly conspicuous, concolorous to light green; cincinni bracts ca. 1.4–5.1–(10.0) × 1.0–1.6 mm, lanceolate to ovate, light green to pink or vinaceous, glandular-pubescent to glabrous, base truncate, margins glabrous, sometimes with a tooth at the base in each side, apex acute; cincinni 1-flowered, patent to erect, straight, peduncle inconspicuous, internodes absent; bracteoles ca. 0.8–1.2 × 0.3–0.6 mm, persistent, triangular, flat, light green to pink, glabrous, base truncate, margins glabrous, apex acute. *Flowers* bisexual or male, enantiostylous, ca. 1.3–2.5 cm diam.; floral buds narrowly ovoid, 5.3–6.2 × 2.6–3.2 mm, light green to pink; pedicels 1.0–5.2 mm long, light green to pink or vinaceous, with glandular to densely glandular, hyaline hairs, deflexed and elongate in fruit; sepals 5.3–8.0 × 1.8–4.7 mm, triangular to ovate-triangular, cucullate, light green to pink to vinaceous, with glandular to densely glandular, hyaline hairs, apex acuminate, margins hyaline light green to hyaline pink or vinaceous; petals equal, 0.8–1.3 × 0.5–0.7 cm, obovate to narrowly obovate, slightly cucullate, white to lilac to purple or mauve, with minute glandular hairs at base on the adaxial surface, base cuneate, margins entire to erose at the apex, apex acute to obtuse; stamens 3, equal, filaments gently curved at the apex, 6.0–9.6 mm long, pale lilac to lilac or purple, with minute glandular, hyaline hairs, anthers elliptic to oblong, 0.9–2.0 × 0.4–0.7 mm, connective purple to bluish purple, anthers sacs lilac to purple, pollen white; staminodes 3, equal, filaments straight, 3.1–5.3 mm long, pale lilac to white, with minute glandular, hyaline hairs, antherodes sagittate, 0.8–2.3 × 0.8–1.1 mm, connective golden yellow, lobes conspicuous, cream-colored to pale yellow; ovary ellipsoid to oblongoid, 1.5–3.5 × 0.7–1.3 mm, 3-locular, light green to green, smooth, with densely glandular, hyaline hairs, style gently curved at the apex, ca. 3.5–8.0 mm, pale lilac to lilac, with minute glandular, hyaline hairs, stigma capitate, lilac to purple. *Capsules* 5.1–9.8 × 3.2–5.0 mm, 3-locular, 3-valved; oblongoid to broadly oblongoid, apiculate due to persistent style, light brown when mature, smooth, with sparse glandular, hyaline hairs, sometimes becoming glabrous with age. *Seeds* 2 per locule, 3.4–4.2 × 1.7–2.1 mm, reniform to broadly ellipsoid, strongly cleft towards the embryotega, ventrally flattened, testa dark brown to greyish brown, sparsely farinose, scrobiculate, with ridges radiating from the embryotega, with a tan appendage that extends ventri-laterally to the embryotega and basally into the hilum; embryotega semilateral, relatively inconspicuous, without a prominent apicule; hilum linear, approximately the same length as the seed, in a shallow depression.

#### Specimens seen.

**BRAZIL. Distrito Federal**: Brasília, immediately N of Brasília, rio Torto, 18 Sep 1965, H.S. Irwin et al. 8425 (NY, RB, US); **Mato Grosso**: Santa Cruz Do Xingu, Parque Estadual do Xingu, limite norte do parque, 6 Mar 2011, D.C. Zappi et al. 3166 (K, RB, UNEMAT); São Félix do Araguaia, estrada entre a vila Pontinópolis e a Serra dos Magalhães, 21 Mar 1997, V.C. Souza et al. 14814 (ESA, RB); Sinop, estrada para Porto dos Gaúchos, ca. 500 km leste do rio Teles Pires, 22 Oct 2004, V.C. Souza 30056 (ESA); Xavantina, Camp B of Base Camp, 10 Jan 1968, D. Philcox & A. Ferreira 3958 (K); loc. cit., 10 km E from base, ca. 270 km from Xavantina, 6 Mar 1968, fl, D.R. Gifford RG76 (K); loc. cit., s.dat., fl., fr., G.R.D. Smith 43 (K); loc. cit., Oct-Nov 1967, fl., J. Ramos & R. Sousa R7 S30 (K); loc. cit., 1 km S of base camp, 14 Mar 1968, D. Philcox & A. Ferreira 4539 (K, NY, P, UB); loc. cit., Xavantina-Cachimbo road, 1 km E of km 244, 15 Mar 1968, D. Philcox & A. Ferreira 4550 (K, NY, P, RB, UB); loc. cit., close to the Xavantina-São Félix do Araguaia road, 11 Apr 1968, J.A. Ratter et al. 992 (K, NY, P, UB); loc. cit., córrego do Porco, 240 km N of Xavantina, 7 May 1968, J.A. Ratter et al. 1339 (K, NY, P, RB, UB); loc. cit., 5 Oct 1968, R.M. Harley 10489 (K, NY, P, RB, UB); loc. cit., 10 Oct 1968, R.M. Harley et al. 10591 (K, NY, P, RB); loc. cit., arredores do acampamento da expedição inglesa até o córrego do Surucucu, 10 Oct 1968, Sidney & Onishi 1356 (RB, UB); **Mato Grosso do Sul**: Indaiá do Sul/Chapéu Azul, cachoeira aos fundos da cidade, 18 Feb 1996, M.R. Pietrobom da Silva et al. 2923 (MBM); Sidrolândia, fazenda Olho D’água, km 392 da Estrada Campo Grande-Sidrolândia, 19 Apr 2013, S.N. Moreira et al. 1451 (BHCB); **Minas Gerais**: Araxá, próximo a Araxá, vale do rio Araguarí, 1 Nov 1970, A.P. Duarte 13912 (HB, MBM). **PARAGUAY. Amambay**: Sierra de Amambay, April 1912–1913, E. Hassler 11347 (BM, K, P).

#### Distribution and habitat.

*Murdannia paraguayensis* occurs in Paraguay and central Brazil, being known from the states of Distrito Federal, Goiás, Mato Grosso, Mato Grosso do Sul and Minas Gerais (Fig. 10). It grows in open flooded grass fields, of the Amazon, Cerrado, Chaco and Pantanal domains.

#### Phenology.

It was found in bloom and fruit throughout the year.

#### Conservation status.

*Murdannia paraguayensis* possesses one of the widest distribution ranges among Neotropical *Murdannia*, with a EOO of ca. 886,876.606 km^2^ and a AOO of ca. 22,500.000 km^2^. Thus, following the IUCN recommendations (IUCN 2001), *Murdannia paraguayensis* should be considered Least Concern.

#### Nomenclatural notes.

When describing *Aneilema paraguayensis*, Chodat (1901) only mentions “Ipé-hu, Oct., 5083”, at the end of his brief diagnosis. According to Stafleu and Cowan (1979), Hassler’s Paraguayan collections are generally housed at G. After consulting several herbaria, we found a specimen at NY herbarium, two specimens at G, and one at BM that matched the protologue. Thus, we selected as the lectotype the specimen at G which shows the typical deflexed pedicel characteristic of this species.

#### Discussion.

*Murdannia paraguayensis* has been historically confused with *Murdannia gardneri* *s.l.*, due to the verticillate cincinni in the inflorescence. For differences between *Murdannia burchellii*, *Murdannia gardneri* and *Murdannia paraguayensis*, see the comments on those species above and Table 1. Despite this historic confusion, *Murdannia paraguayensis* is morphologically very similar to *Murdannia engelsii*, due to its petals, androecium and gynoecium with glandular hairs, pedicels deflexed postanthesis and in fruit, and capitate stigma. Nevertheless, *Murdannia paraguayensis* can be differentiated by its erect habit (*vs.* trailing in *Murdannia engelsii*), leaves spirally-alternate (*vs.* distichously-alternate), much larger inflorescences with several whorls of cincinni (*vs.* consisting of a solitary cincinnus), peduncle solely with glandular hairs (vs. with a mixture of eglandular and glandular hairs), cincinni 1-flowered (*vs.* 2–7-flowered), capsules oblongoid to broadly oblongoid (*vs.* broadly ovoid to broadly ellipsoid), and locules 2-seeded (*vs.* locules 1-seeded).

The specimen *H.S. Irwin et al. 8425* looks very distinctive from the other analyzed specimens due to its: apparently creeping habit, leaves distichously-alternate at apex, sheaths with a few scattered long glandular hairs, blades with strongly undulate margins, short congested inflorescence, and very short pedicels. Nevertheless, it possesses the same inflorescence architecture, capsules with glandular hairs, and 2-seeded locules. We believe that the blades with strongly undulate margins may be a result of the drying process. Thus, we consider that these collections don’t merit any taxonomic recognition.

### 6. Murdannia schomburgkiana

Taxon classificationPlantaeCommelinalesCommelinaceae

(Kunth) G.Brückn., Nat. Pflanzenfam. (ed. 2)15a: 173. 1930.

Figs 7 , 10

Phaeneilema schomburgkiana (Kunth) G.Brückn., Notizbl. Bot. Gart. Berlin–Dahlem 10 (91): 56. 1927.
Aneilema schomburgkianum Kunth, Enum. Pl. 4: 661. 1843. Lectotype (designated here): GUYANA. s.loc., fl., fr., Oct 1841, R.H. Schomburgk 842 (B barcode B100367820!; isolectotypes: 2 ex BM not found, G barcodes G00176335!, G00176336!, G00176337!, P barcodes P02088026!, P02088027!, TCD barcode TCD0008088!).

#### Description.

*Herbs* ca. 30.0–65.0 cm tall, perennial, rhizomatous with a definite base, terrestrial to paludal to rooted emergent in open flooded savannas. *Roots* tuberous, thick and fusiform, medium to dark brown, densely to sparsely pilose with medium to dark brown hairs, emerging from the short rhizome and from the basal nodes. *Rhizomes* short, brown, buried in the sand or soil. *Stems* erect, succulent, unbranched; internodes 1.0–11.5 cm long, green to vinaceous, glabrous, sometimes with a line of hyaline eglandular hairs opposite the leaf above. *Leaves* spirally-alternate, evenly distributed along the stems, sessile, the distal ones gradually smaller than the basal ones; sheaths 0.8–2.2 cm long, green to vinaceous, glabrous, with a line of hyaline, eglandular hairs opposite the leaf above; lamina 2.2–14 × 0.4–1.0 cm, membranous to succulent, canaliculate, slightly falcate, green on both sides, glaucous, drying olive-green to light green on both sides, linear-elliptic to linear-lanceolate, glabrous, base truncate to rounded, margins light green, glabrous, apex acuminate; midvein slightly conspicuous to inconspicuous, slightly impressed adaxially, slightly obtuse abaxially, secondary veins 2–3–(4) pairs, adaxially inconspicuous to slightly conspicuous, light green, abaxially slightly conspicuous. *Inflorescences* 1–4, terminal or axillary in the uppermost nodes, fascicle-like, composed of 1–2–(3) verticillate cincinni; peduncles absent; basal bract inconspicuous; cincinni bracts 1.6–1.8 × 0.3–0.4 cm, tubular, amplexicaul; cincinni 1-flowered, erect, straight, peduncle 1.0–1.9 cm long, light green to pink or vinaceous, glabrous; bracteoles inconspicuous, generally caducous. *Flowers* bisexual or male, actinomorphic, ca. 1.3–2.3 cm diam.; floral buds ellipsoid, 5.0–5.8 × 1.5–1.8 mm, light green to pink; pedicels 0.6–1.1 cm long, light green to pink to vinaceous, glabrous, erect and elongate in fruit; sepals 6.5–10.0 × 3.2–4.1 mm, triangular to ovate-triangular, cucullate, pink to pinkish brown, glabrous, apex acute, margins hyaline pink to hyaline vinaceous; petals equal, 0.8–1.3 × 0.8–1.0 cm, obovate to broadly obovate, slightly cucullate, lilac to purple, medially bearded with lilac to purple, moniliform hairs on the adaxial surface, base cuneate, margins entire, apex acute to obtuse; stamens 3, equal, filaments gently curved at the apex, 4.4–5.2 mm long, lilac to purple, densely bearded with moniliform, lilac to purple hairs, hairs slightly shorter than the filaments, anthers elliptic to oblong, 1.7–2.4 × 0.6–1.0 mm, connective brown, anthers sacs brownish lilac, pollen brownish lilac; staminodes 3, equal, filaments straight, 4.1–5.0 mm long, pale lilac to lilac, densely bearded with moniliform, lilac to purple hairs, hairs slightly shorter than the filaments, antherodes hastate, 0.9–1.7 × 1.3–1.7 mm, connective golden yellow, lobes conspicuous, cream-colored to pale yellow; ovary ellipsoid to oblongoid, 1.9–3.1 × 0.7–1.3 mm, 3-locular, light green to green, smooth, glabrous, style gently curved at the apex, ca. 4.1–5.4 mm, lilac to purple, stigma capitate, lilac to purple. *Capsules* 5.9–8.5 × 2.8–4.6 mm, 3-locular, 3-valved, oblongoid to broadly oblongoid, apiculate due to persistent style, light brown when mature, smooth, glabrous. *Seeds* (immature) 6 per locule, 2.7–3.3 × 2.6–3.1 mm, cuboid to polygonal, slightly cleft towards the embryotega, testa dark brown to greyish brown, densely farinose, scrobiculate, with ridges radiating from the embryotega; embryotega semilateral, relatively inconspicuous, without a prominent apicule, generally covered by a cream farina; hilum linear, ½ the length of the seed or smaller, on a weak ridge.

#### Specimens seen.

**BRAZIL. Amazonas**: Provincia do Rio Negro, Rio Madeira, fl., s.dat., s.leg. s.n. (P barcode P03653202); s.loc., fl., Oct 1894, A.R. Ferreira 755 (K). **GUYANA. Rupununi District**: foot of Mount Shiriri, fl., 19 Jun 1995, M.J. Jansen-Jacobs et al. 4175 (P, U, US); loc. cit., Manari, Takatu river, fl., 5 Aug 1995, M.J. Jansen-Jacobs et al. 4764 (U, US); loc. cit., upper Rupununi river, fl., *Appun 2361* (K).

#### Distribution and habitat.

*Murdannia schomburgkiana* is known from only four collections from Guyana (including the type) and perhaps only one collection from Brazil (in the state of Amazonas) (Fig. 10). It grows in open flooded grass fields and savannas in the Amazon domain. The distance between the Rio Madeira specimen and the other specimens collected in Guyana, make clear how poorly collected this species is. It is widely possible that field trips focusing on the group or in the white sand formations in the state of Amazonas will fill this distribution gap.

It is interesting to highlight that both specimens from Brazil might represent different sheets of the same collection. Firstly, it is known that Dr. Alexandre Ferreira collected exclusively in Brazilian territory. Thus, despite the locality not being clearly stated in the label of the specimen at Kew, this is the only possible option. Secondly, the specimen at Paris was collected in Brazil, Provincia Rio Negro, at the margins of Rio Madeira (currently state of Amazonas). This was one of the most important areas collected by Ferreira, during his philosophical travels, and probably the longest part of this fieldtrip. Also, it is widely known that many specimens collected by Friar Vellozo, Dr. Vellozo de Miranda and Dr. Alexandre Ferreira, were taken from Lisbon to Paris, during the Napoleonic Wars. Finally, the labels of both specimens possess complementary information, where the locality in the label of the Paris’ specimen is one of locations where Ferreira collected, and the date is congruent with this specific fieldtrip. Moreover, the specimens on both sheets are very similar in appearance.

#### Phenology.

It was found in bloom from June to October, and in fruit in October.

#### Conservation status.

*Murdannia schomburgkiana* is only known from five (or at most six) collections, including the type species. Furthermore, the last known collections for this species are 11 years old, and the AOO of *Murdannia schomburgkiana* is of only ca. 12.000 km^2^. Following the IUCN recommendations (IUCN 2001), *Murdannia schomburgkiana* should be considered Endangered [EN, B1a+C2a(ii)+D1].

#### Nomenclatural notes.

When describing *Aneilema schomburgkiana*, Kunth (1843) mentions “Rob. Schomburgk misit sub. no. 842”. According to Stafleu and Cowan (1985), Robert Schomburgk’s collections are generally housed at BM or K. Despite having found two specimens at BM, the specimen at B (B100367820) possesses the annotation “Ex. herb. Kunth misit. 1841.”, made in Kunth’s handwriting and matching the protologue, and it is widely known that Kunth’s herbarium was part of B (Stafleu and Cowan 1979). Thus, it was the obvious choice for a lectotype. The two sheets at BM were observed and described in detail by one of us (RBF) in 1993. However, they were not photographed when other types at BM were photographed, and the specimens cannot currently be located. If found they should be treated as isolectotypes.

#### Discussion.

*Murdannia schomburgkiana* can be easily confused with *Murdannia semifoliata* (C.B.Clarke) G.Brückn., due to their tuberous roots, reduced inflorescences enclosed by the leaf-sheaths, cincinni bracts tubular, petals medially bearded with moniliform hairs on the adaxial surface, filaments densely bearded with moniliform hairs, the number of seeds per locule of the capsule, and seed morphology. Their petals medially bearded with moniliform hairs on the adaxial surface, are quite unique within *Murdannia*. As aforementioned, this character is otherwise only known in Commelinaceae in *Murdannia simplex* (in which the hairs are tiny and only present at the petal, being fundamentally different), and in the distantly related genera *Cochliostema* Lem. and *Geogenanthus* Ule (Tribe Tradescantieae, subtribe Dichorisandrinae; Hardy and Faden 2004; Pellegrini in press). Nevertheless, the distribution of both species does not overlap and they grow in different environments (white sand formations *vs.* flooded grass fields). *Murdannia schomburgkiana* can be differentiated by its 2.2–13.6 cm long blades of the leaves bearing inflorescences (*vs.* 0.2–1.8 cm long), leaf-blades margins glabrous (*vs.* ciliate), cincinni bracts 1.6–1.8 cm long (vs. 0.4–1.3 cm long), and brown anthers (*vs.* purple) (Table 1).

Despite the few collections known for this species, it is the authors’ opinion that the morphological, geographical and environmental factors are enough to differentiate both species. *Murdannia schomburgkiana* and *Murdannia semifoliata* are very similar to each other, and quite distinct from the remaining Neotropical species of the genus. They are morphologically similar to some Asian and African species with fascicle-like, mainly axillary inflorescences, and 1-flowered cincinni, such as *Murdannia axillaris* and *Murdannia triquetra*.

### 7. Murdannia semifoliata

Taxon classificationPlantaeCommelinalesCommelinaceae

(C.B.Clarke) G.Brückn., Nat. Pflanzenfam. (ed. 2)15a: 173. 1930.

Figs 8 , 10

Phaeneilema semifoliata (C.B.Clarke) G.Brückn., Notizbl. Bot. Gart. Berlin–Dahlem 10 (91): 56. 1927.
Aneilema semifoliatum C.B.Clarke, C.B.Clarke in Moore, Trans. Linn. Soc. London, Bot. 4: 498. 1895. Lectotype (designated here): BRAZIL. Mato Grosso: Santa Cruz [do Xingú], fl., Oct 1891–1892, S.M. Moore 541 (BM barcode BM000938202!; isolectotypes: B barcode B100367821!, NY barcode NY00247404!).

#### Description.

*Herbs* ca. 20.0–70.0 cm tall, perennial, rhizomatous with a definite base, terrestrial to paludal to rooted emergent in open flooded fields. *Roots* tuberous, thick and fusiform, medium to dark brown, densely to sparsely pilose with medium to dark brown hairs, emerging from the short rhizome and from the basal nodes. *Rhizomes* short, brown, buried in the sand or soil. *Stems* erect, succulent, unbranched; internodes 1.2–13.3 cm long, green to vinaceous, glabrous, with a line of hyaline, eglandular hairs opposite to the leaf above. *Leaves* spirally-alternate, evenly distributed along the stems, the distal ones much smaller than the basal ones (which are generally bladeless sheaths with lamina no longer than 1.8 cm); sheaths 0.5–2.3 cm long, green to vinaceous, glabrous, with a line of hyaline, eglandular hairs opposite to the leaf above, margins setose to ciliate; lamina 0.2–8.9 × 0.2–0.7 cm, succulent, canaliculate, slightly falcate, green on both sides, glaucous, drying olive-green on both sides, linear-triangular to triangular, glabrous, base truncate, margins light green, setose at the base, ciliate at the middle, glabrous at the apex, apex acuminate; midvein inconspicuous on both sides, rarely slightly obtuse abaxially, secondary veins inconspicuous. *Inflorescences* (1–)2–6, terminal and axillary from the uppermost nodes, fascicle-like, composed of 1–2–(3) verticillate cincinni; peduncles absent; basal bract inconspicuous; cincinni bracts 0.4–1.3 × 0.1–0.3 cm, tubular, amplexicaul; cincinni 1-flowered, erect, straight, peduncle 0.8–4.2 mm long, light green to pink to vinaceous, glabrous, internodes inconspicuous; bracteoles inconspicuous, generally caducous. *Flowers* bisexual or male, actinomorphic, ca. 0.6–2.3 cm diam.; floral buds ellipsoid, 4.9–7.2 × 1.7–2.2 mm, light green to pink; pedicels 1.4–1.1 mm long, light green to pink to vinaceous, glabrous, erect and elongate in fruit; sepals 4.8–8.0 × 1.8–3.3 mm, triangular to ovate-triangular, cucullate, pink to pinkish brown, glabrous, apex acute, margins hyaline pink to hyaline vinaceous; petals equal, 0.5–1.2 × 0.3–0.8 cm, obovate, slightly cucullate, lilac to purple or mauve, rarely white, medially bearded with moniliform hairs on the adaxial surface, hairs lilac to purple, base cuneate, margins entire, apex acute to obtuse; stamens 3, equal, filaments gently curved at the apex, 3.2–5.0 mm long, lilac to purple, densely bearded with moniliform, lilac to purple hairs, hairs slightly shorter than the filaments, anthers linear-oblong to oblong, 2.0–3.5 × 0.4–0.7 mm, connective purple, anthers sacs lilac to purple, pollen lilac; staminodes 3, equal, filaments straight, 3.1–4.3 mm long, pale lilac to lilac, densely bearded with moniliform, lilac to purple hairs, hairs slightly shorter than the filaments, antherodes hastate, 0.7–2.0 × 0.5–1.2 mm, connective golden yellow, lobes conspicuous, cream-colored to pale yellow; ovary ellipsoid to oblongoid, 1.5–3.3 × 0.5–1.0 mm, 3-locular, light green to green, smooth, glabrous, style gently curved at the apex, ca. 3.2–4.5 mm, lilac to purple, stigma capitate, lilac to purple. *Capsules* 5.8–1.2 × 3.3–5.6 mm, 3-locular, 3-valved; oblongoid to broadly oblongoid, apiculate due to persistent style, light brown when mature, smooth, glabrous. *Seeds* 6 per locule, 2.2–3.1 × 2.0–2.8 mm, cuboid to polygonal, slightly cleft towards the embryotega, testa dark brown to greyish brown, densely farinose, scrobiculate, with ridges radiating from the embryotega; embryotega semilateral, relatively inconspicuous, without a prominent apicule, generally covered by a cream farina; hilum linear, less than ½ the length of the seed, on a weak ridge.

#### Specimens seen.

**BOLIVIA. Santa Cruz**: San Ignacio de Velasco, Oct 1958, M. Cardenas 5629 (BOLV, US). **BRAZIL. Mato Grosso**: Bananalzinho, Nov 1914, J.G. Kuhlmann 89 (R, SP); Braco, rio Arinos, 26 Sep 1943, J.T. Baldwin Jr. 3097 (US); Cuiabá, entre Cuiabá e Goyaz, Nov–Dec 1844, M.A. Weddell 3018 (P); loc. cit., rodovia MT-364, 35 km S de Cuiabá, 13 Nov 1975, G. Hatschbach 37491 (K, MBM); Nova Olímpia, Chapada dos Guimarães, 10 Oct 1995, J.H.A. Dutilh 199 (UEC); Poconé, 50 km S of Poconé on Transpantaneira highway to Porto Jofre, 27 Oct 1985, W. Thomas et al. 4641 (INPA, NY, US); loc. cit., highway Poconé-Porto Cercado, ca. km 21, 17 Feb 1992, M. Schessl 100/1-10 (UFMT, US); loc. cit., about 21 km S of Poconé, 7 Oct 1992, M. Schessl 071092-1-1 (UFMT, US); loc. cit., fazenda Ronco Bugiu, ca. 6–8 km à esquerda da rodovia Transpantaneira Poconé-P. Jofre, km 36, 31 Oct 1992, A.L. Prado et al. 3218 (HURB, UEC, UFMT); loc. cit., 22 Nov 1992, A.L. Prado et al. 2736 (HURB, UEC, UFMT); Rosário Oeste, ca. 2 km de Marzagão em direção à Planalto da Serra, 7 Oct 1997, V.C. Souza et al. 20255 (ESA, UFMT, UEC); Santo Antônio de Leverger, Barão do Melaço, km 30 of Leverger highway, 5 Nov 1991, M. Schessl 2421 (CH, UFMT, US); **Mato Grosso do Sul**: Aquidauana, entre as fazendas São Salvador e Costa Rica, 19 Nov 1995, A. Pott et al. 7628 (CGMS, CPAP, US); loc. cit., rodovia Taunay, fazenda Santa Cruz, próximo da aldeia indígena Ipegue, 20 Nov 2002, G. Hatschbach et al. 74377 (MBM).

#### Distribution and habitat.

*Murdannia semifoliata* occurs mainly in Brazil (in the states of Mato Grosso and Mato Grosso do Sul) and in Bolivia (Fig. 10). It grows in open flooded grass fields in the Amazon, Cerrado and Chaco domains.

#### Phenology.

It was found in bloom and fruit from September to February.

#### Conservation status.

*Murdannia semifoliata* possesses a EOO of ca. 298,091.226 km^2^ and a AOO of ca. 22,500.000 km^2^. Despite the relatively great number of collections, most of them are in the state of Mato Grosso, with only one known collection on the state of Mato Grosso do Sul and another one from Bolivia. This whole region is under great treat due to the constant deforestation for cattle ranching. Thus, we believe that following the IUCN recommendations (IUCN 2001), *Murdannia semifoliata* should be considered Nearly Threatened.

#### Nomenclatural notes.

When describing *Aneilema semifoliatum*, Clarke (1895) mentions “Crescit ad Santa Cruz, ubi mens. Oct. floret. (N. 541)”. The specimen at BM matched the protologue perfectly. Furthermore, it possesses a detailed description and was identified by Clark himself. Thus, it is here designated as the lectotype of *Aneilema semifoliatum*.

#### Discussion.

*Murdannia semifoliata*, as aforementioned, is morphologically similar to *Murdannia schomburgkiana*. They share a peculiar vegetative morphology, inflorescence architecture, and petals medially bearded with moniliform hairs on the adaxial surface, not similar to any other Neotropical species. *Murdannia semifoliata* is especially distinctive due to its extremely reduced blades of the leaves bearing inflorescences, produced during the flowering period (Table 1). In most individuals, the blades are so reduced that the whole plant seems to be aphyllous. Furthermore, *Murdannia semifoliata* and *Murdannia schomburgkiana* are the only Neotropical species to possess more than two seeds per locule, which gives the seeds a peculiar cuboid to polygonal shape.

### 8. Murdannia aff. triquetra

Taxon classificationPlantaeCommelinalesCommelinaceae

(Wall. ex C.B.Clarke) G.Brückn., Nat. Pfl.-Syst. (ed. 2) 15a: 173. 1930.

Fig. 9

Phaeneilema triquetrum (Wall. ex C.B.Clarke) G.Brückn., Notizbl. Bot. Gart. Berlin–Dahlem 10: 56. 1927.
Aneilema triquetra Wall. ex C.B.Clarke, Monogr. Phan. 3: 208. 1881. Lectotype (designated by Ancy et al. 2015): BANGLADESH. India Orientalis, in Prov. Sylhet, fl., fr., s.dat., N. Wallich 5220 (B barcode B100367814!: isolectotypes: E barcode E00393352!, GDC barcode GDC00489348!; K n.v.).

#### Diagnosis.

*Herbs* ca. 10.0–20.0 cm tall, annual, without a definite base, rooted emergent in flooded fields. *Roots* thin, fibrous, medium to dark brown, densely to sparsely pilose with medium to dark brown hairs, emerging from the basalmost nodes. *Rhizomes* absent. *Stems* trailing, floating on water with ascending apex, succulent, densely branched at the base, glabrous or with minute eglandular hairs. *Leaves* spirally-alternate, evenly distributed along the stems; sheaths 0.7–1.0 cm long, glabrous; lamina 2.0–4.5 × 0.6 cm, narrowly lanceolate to lanceolate-oblong, glabrous membranous, slightly canaliculate, green on both sides, base rounded to amplexicaul, margins glabrous, sometimes undulate, apex acute to acuminate. *Inflorescences* 1–3, terminal or axillary in the distalmost (up to 4) nodes, fascicle-like, sessile, enclosed by the leaf-sheaths, composed of 1–2–(3) verticillate cincinni; peduncle absent; basal bract inconspicuous; cincinni bracts absent; cincinni 1-flowered, erect, straight, peduncle ca. 3.0 mm long, glabrous, internodes inconspicuous; bracteoles absent. *Flowers* male or bisexual, actinomorphic, barely exserted from the sheath; floral buds ellipsoid, light green; pedicels ca. 3 mm long, erect and elongate in fruit; sepals 4.0–5.5 mm long, linear-elliptic, cucullate, light green to pale pink, glabrous; petals equal, elliptic, slightly cucullate, white to pale lilac or pale pink, glabrous; androecium not determinable; ovary ellipsoid, tapering into the style, 3-locular, light green, smooth, glabrous, style straight, 1.7 mm long, glabrous, stigma capitate. *Capsules* 4.5–5.5 × 2.0–2.5 mm, oblongoid to ellipsoid, 3-locular, 3-valved, apiculate due to persistent style, light brown when mature, smooth, glabrous, locules 3-seeded (only 1 counted). *Seeds* (only 1 mature seed seen) transversely ellipsoid, ca. 1.5 × 0.9 mm, testa brown, with deep dorsal pits and longitudinal furrows, farinose only around the embryotega, appendage absent; embryotega lateral, inconspicuous, without a prominent apicule; hilum linear, less than ½ the length of the seed, borne on a ridge.

#### Specimen seen.

VENEZUELA. Tachira. Distr. Liberatador: 10 km S of El Piñal, 71°55'W, 7°27'N, alt. 250 m, 7 Nov. 1982, G. Davidse & A. C. González 21663 (US).

#### Distribution and habitat.

Known for certain only from this collection. The general habitat was recorded as “partially inundated forest remnant with slow stream and pools of standing water” and for this collection as “stems floating in pool of creek.” A photograph of a plant from Colombia, which may or may not be the same species, was sent to the first author, but without a corroborating specimen, so it has not been considered for this description. However, we have illustrated it in Fig. 9 to encourage collectors to look for it.

The *Murdannia keisak* complex is widespread in Asia, ranging from India to China and Japan, growing in flooded grasslands and disturbed areas. In South America, it is known from only two collections, one from Venezuela and one from Colombia. Unfortunately, it seems that the specimen from Colombia went astray during shipping, since it was never received by the first author.

#### Phenology.

It was found in bloom and fruit in November.

#### Conservation status.

Following the IUCN recommendations (IUCN 2001), this species should be considered Data Deficient. Correspondence by the second author with the collector Gerrit Davidse, indicated that this was not a disturbed habitat in which one would expect to find introduced weeds. However, the habitat was under great pressure and possibly no longer exists.

#### Nomenclatural notes.

Nandikar and Gurav (2015) designated the specimen at CAL (CAL0000025807) as the lectotype for *Aneilema triquetrum*. Nevertheless, after analyzing the specimen, comparing it to the protologue and to the remaining specimens, it became clear that the specimen at CAL is not conspecific to the specimens at B, E and GDC. Ancy et al. (2015), unaware of the article published just few months earlier by Nandikar and Gurav (2015), designate the specimen at B (B100367814) as the lectotype for *Aneilema triquetrum*. Their choice matches perfectly the protologue, and thus should be followed instead of the lectotypification made by Nandikar and Gurav (2015). Nonetheless, if ever found, the specimen at K would make a much better choice of a lectotype. At the time of the description of *Aneilema triquetrum* and the completion of his monograph (i.e. 1881), Clarke was working at K, and would had access to a possible specimen in the Wallich Herbarium, housed at Kew.

#### Discussion.

This is a widely distributed species complex, being very common and well collected in Asia. Nevertheless, the morphologic limits between *Murdannia keisak* and *Murdannia triquetra*, as well as the application of these names, varies greatly according to each author. In Flora of China (Hong and DeFilipps 2000), both species are accepted, although somewhat tentatively, and are separated by the length of the sepals, shape and size of the capsule, and number and shape of the seeds. The authors also state that the morphologic differences seem to be associated with the geographic distribution of the taxa. Nevertheless, both descriptions overlap with the description presented by Faden (2000) for *Murdannia keisak*, in North America. Ancy (2014), in her unpublished Ph.D. thesis, presents a thorough taxonomic account on *Murdannia* from India. Her description of *Murdannia triquetra* matches very closely the two specimens known for South America, in sepal, petal and fruit morphology. Nonetheless, Ancy (2014) describes the filaments as being glabrous, contrary to the bearded filaments known for the South American specimens. The author also omits the description of the antherodes, which in the South American specimens are yellow and cordate. Nevertheless, some young flower buds dissected by the second author lacked hairs on the filaments of the stamens and completely lacked staminodes, but that might have been a developmental stage and thus may not be a discrepancy. This could be related to the extremely immature state of the dissected buds, and could explain the discrepancy of our description and the description presented by Ancy (2014). Nandikar and Gurav (2015) published a second account on the Indian species of *Murdannia*. In their treatment, *Murdannia triquetra* differs greatly from the South American specimens. However, it matches very closely the description presented by Hong and DeFilipps (2000), Faden (2000) and Chowdhury et al. (2015) for *Murdannia keisak*. In these descriptions, the antherodes are described as sagittate and ranging from lilac to purple, and clearly do not match the South American specimens.

It is the authors opinion that a study focusing on the specific boundaries between these taxa is necessary. Nevertheless, since this species complex is only invasive in the New World, we also believe that the required investigation should be carried out in the plants native range. It is also possible that these Neotropical collections represent a distinct taxon, not closely related to the other native South American species. But a much better South American sampling for comparison and a much more detailed would be required. Field work, better sampling of herbaria specimens, detailed study of reproductive morphology, analysis of the protologues, and population studies might shed a light on the issue.

## Conclusions

Neotropical *Murdannia* is represented by six native species confined to South America, mostly in Brazil. The species can be distinguished from one another by growth habit, branching pattern of the stems, phyllotaxy, indumentum type, inflorescence morphology, indumentum on the petals, androecium and gynoecium, capsule morphology, seed shape, and by the ornamentation of the testa. Two invasive species, native to Asia, are found in the Neotropics. *Murdannia* aff. *triquetra* is recorded for the first time in South America. Despite being rarely collected, the known South American populations seem to be well-established and should be monitored to avoid the dispersal of yet another invasive species of Commelinaceae. It may be mentioned, for the sake of completeness, that the only other *Murdannia* species recorded from the Western Hemisphere is the Asian taxon *Murdannia spirata* (L.) G.Brückn., which in naturalized in southern Florida, United States (Faden 2000).

Despite being seldom collected, Neotropical *Murdannia* are generally described in labels as forming large populations. It is possible that the lack of collections for the group is connected to: (1) the difficulty to access the areas where they occur (e.g. Amazonian river banks); (2) general neglect of aquatic flora, due to logistic difficulties in field work; (3) the difficulty to preserve Commelinaceae flowers in dried specimens, discouraging botanists to collect them; (4) and lack of field work focusing on herbaceous plants. The authors hope that the present work will encourage field workers to collect Commelinaceae specimens in the Amazon, Cerrado, Chaco and Pantanal domains. Furthermore, the increase of collections will enable researchers to monitor these species’ populations in order to update and provide more precise conservation assessments for them, and monitor the need for biological control of the known invasive species.

Although several studies focusing on morphology, anatomy and cytology of *Murdannia* are available in the literature, no comprehensive phylogenetic study has been presented up to date. Burns et al. (2011) were the first to sample more than one species of *Murdannia* in a phylogenetic analysis. However, all of the five sampled species were Asian and none represented the type-species. Thus, the monophyly of *Murdannia* is still to be tested in future studies. Ancy (2014) presented a morphological phylogeny, sampling exclusively the species native to India. In her analysis, the clades are supported by characters like inflorescence architecture, and androecium, capsule and seed morphology. As aforementioned, the Neotropical species of *Murdannia* are extremely peculiar in a considerable number of morphological characters, and nothing is known regarding their phylogenetic relationships, anatomy or even their cytology. Thus, three important questions about the Neotropical species are: (1) how are they related to one another; (2) what is the relationship between the Neotropical species and the rest of the genus; and (3) how many dispersal events the Neotropical lineages of *Murdannia* would represent. In a more general sense, it would also be important to understand the evolution of morphological characters in the genus on a phylogenetic framework, such as the inflorescence and androecium morphology.

## Supplementary Material

XML Treatment for Murdannia

XML Treatment for Murdannia burchellii

XML Treatment for Murdannia engelsii

XML Treatment for Murdannia gardneri

XML Treatment for Murdannia nudiflora

XML Treatment for Murdannia paraguayensis

XML Treatment for Murdannia schomburgkiana

XML Treatment for Murdannia semifoliata

XML Treatment for Murdannia aff. triquetra
